# Effectiveness of pharmaceutical care interventions amid pharmacist role transformation in China: a meta-analysis

**DOI:** 10.3389/fpubh.2026.1860119

**Published:** 2026-06-19

**Authors:** Pengyu Zhao, Shuai Geng

**Affiliations:** 1 Department of Education, Chinese Academy of Medical Sciences, Peking Union Medical College Hospital, Beijing, China; 2 Department of Pharmacy, The Ninth Medical Center of PLA General Hospital, Beijing, China

**Keywords:** antimicrobial agents, meta-analysis, pharmaceutical care, pharmacist, rational drug use, role transformation

## Abstract

**Objective:**

To evaluate the effectiveness of pharmaceutical care delivered by Chinese pharmacists during their professional role transformation, including prescription review, medication counseling, prescription comment, and the value of pharmacist interventions in multidisciplinary team settings, with the aim of providing evidence-based support for advancing the transformation and optimizing service models of the pharmacy workforce in China.

**Methods:**

We searched PubMed, Embase, Cochrane Library, Web of Science, CBM, CNKI, Wanfang, and VIP from inception to April 2026. RCTs and NRSIs examining pharmacist-delivered pharmaceutical care in Chinese institutions were included. Risk of bias was assessed with Cochrane ROB and ROBINS-I. Meta-analyses used odds ratios (ORs) and 95% CIs.

**Results:**

Thirty-two studies (5 RCTs, 27 NRSIs) were included. Pharmacist interventions were associated with increased single-agent antimicrobial use (OR 3.13, 95%CI 2.49–3.92) and rational prescriptions (OR 2.97, 95%CI 2.30–3.85), and reduced combination therapy, irrational prescriptions (OR 0.22, 95%CI 0.17–0.28), as well as reduced irrational prescribing in intravenous admixture centers and outpatient settings (non-standard: OR 0.46, 95%CI 0.43–0.49; inappropriate: OR 0.51, 95%CI 0.42–0.62; exceptional: OR 0.26, 95%CI 0.08–0.84). Heterogeneity was substantial (*I*^2^ 0–96%), limiting precision.

**Conclusion:**

Pharmacist-led interventions are associated with improved prescribing process indicators in China. However, the evidence is limited by few RCTs, predominance of NRSIs with moderate/high bias, and high heterogeneity. Findings are exploratory and hypothesis-generating. High-quality RCTs with patient-centered outcomes are needed.

## 1 Introduction

Pharmacists’ roles are shifting from drug dispensing to patient-centered care. Since Hepler and Strand introduced pharmaceutical care in 1990, the profession has moved toward direct patient responsibility for therapeutic outcomes (1). The International Pharmaceutical Federation and the World Health Organization have advocated for pharmacists as medication therapy managers. In developed countries, clinical pharmacists are integrated into multidisciplinary teams, reducing hospital readmissions and emergency visits (2).

To clarify key concepts: Pharmaceutical care is patient-centered responsibility for therapy outcomes (1); clinical pharmacy services include medication review, monitoring, and multidisciplinary collaboration; prescription review verifies prescription appropriateness before dispensing; and antimicrobial stewardship optimizes antimicrobial use to combat resistance. In China, pharmacist role transformation increasingly incorporates these functions. This meta-analysis evaluates pharmaceutical care interventions, with sub-analyses focusing on prescription review, antimicrobial stewardship, and other clinical pharmacy services.

The transformation of China‘s pharmacy workforce began relatively late. In 2005, the Chinese Hospital Association launched a clinical pharmacist training program that has since trained more than 10,000 clinical pharmacists across 17 specialties, including respiratory medicine, infectious diseases, and intensive care. In China, the pharmacist role transformation began later. A national clinical pharmacist training program launched in 2005 has trained over 10,000 clinical pharmacists. National policies such as the “Opinions on Accelerating High-Quality Development of Pharmaceutical Services” have called for transforming pharmaceutical service models and strengthening clinical pharmacist deployment (3). However, implementation faces challenges, including weak incentives, poor multidisciplinary collaboration, and incomplete information systems. Many hospital pharmacists still perform traditional dispensing roles. Qualitative studies have identified ambiguous role definitions and insufficient health economics training as barriers (4). Despite evidence that pharmacist involvement can reduce hospital costs (5), systematic evaluations of China‘s pharmacist transformation are lacking. Given the high prevalence of inappropriate antimicrobial use in China and the national priority to combat antimicrobial resistance, most pharmacist intervention studies have targeted antimicrobial prescribing. Hence, this meta-analysis prioritizes antimicrobial prescription outcomes, while also covering other pharmaceutical care services.

Numerous systematic reviews have shown that pharmacist interventions improve medication adherence, reduce adverse events, and optimize clinical outcomes. For example, pharmacist-led care improved anticoagulation appropriateness (OR 3.43) and reduced bleeding events by 25% (6), and shortened hospital stays in perioperative settings (7). Meta-analyses also demonstrate improved adherence in chronic diseases (8). Thus, pharmacist interventions are recognized as effective means to promote rational drug use and control costs. However, there is still a lack of systematic synthesis and evaluation of the effectiveness of pharmaceutical care following pharmacist role transformation in China. In particular, during the country’s transitional period of pharmacist role change, the service outcomes across different practice settings (e.g., outpatient pharmacies, intravenous admixture service centers) and under various intervention models have not yet been systematically integrated and assessed.

Therefore, this meta-analysis systematically evaluates the process-oriented effectiveness of pharmaceutical care delivered by Chinese pharmacists undergoing role transformation, covering prescription review, medication counseling, and multidisciplinary care, to provide evidence-based support for advancing pharmacy workforce transformation in China. Clinical outcomes are not assessed.

## 2 Materials and methods

### 2.1 Literature search method and strategy

We systematically searched the following electronic databases from their inception to April 30, 2026: PubMed, Embase, Cochrane Library, Web of Science, Chinese Biomedical Literature Database (CBM), China National Knowledge Infrastructure (CNKI), Wanfang Data, and VIP Chinese Science and Technology Periodical Database. The search was restricted to articles published in English or Chinese because all eligible studies originated from mainland China and the relevant literature is predominantly available in these two languages.

Search strategy for English databases (exemplified by PubMed):

The following search string was used in PubMed, combining MeSH terms and free words with Boolean operators: (“pharmacists”[MeSH Terms] OR “pharmacist”[Title/Abstract] OR “pharmacists”[Title/Abstract] OR “clinical pharmacist”[Title/Abstract]) AND (“pharmaceutical care”[MeSH Terms] OR “pharmaceutical care”[Title/Abstract] OR “pharmaceutical practice”[Title/Abstract] OR “pharmaceutical service”[Title/Abstract] OR “pharmacy intervention”[Title/Abstract] OR “prescription review”[Title/Abstract] OR “medication counseling”[Title/Abstract]).

This search string was adapted for Embase, Cochrane Library, and Web of Science using equivalent Emtree terms or relevant subject headings, while maintaining the same Boolean logic and field restrictions.

Search strategy for Chinese databases (exemplified by CNKI):

For CNKI, Wanfang Data, VIP, and CBM, we used the following search terms with Boolean operators in Chinese:

(Pharmacist OR Clinical Pharmacist OR Pharmacist) AND (Pharmaceutical Care OR Pharmaceutical Monitoring OR Pharmaceutical Intervention OR Prescription Review OR Medication Guidance OR Prescription Comment OR Rational Drug Use)

The search was performed in titles, abstracts, and keywords. The exact search strings for each Chinese database are available from the corresponding author upon request.

Manual and supplementary searches:

In addition to the electronic database searches, we manually screened the reference lists of all included studies and relevant review articles to identify any additional eligible studies not captured by the electronic searches. We also performed a gray literature search using the Google Scholar platform (first 200 results) with the same keyword combinations, but no additional eligible studies were identified.

The complete search strategies for all databases, including the date of the last search (April 30, 2026), are reported following PRISMA 2020 recommendations in Supplementary Table S1.

### 2.2 Eligibility criteria

Inclusion criteria ① Studies that examined pharmaceutical care proactively and systematically delivered by pharmacists in Chinese healthcare institutions as part of their role transformation. The scope of pharmaceutical care included, but was not limited to: prescription appropriateness review, prescription comment and feedback, patient-oriented medication instruction and education, professional medication counseling, and adverse drug reaction monitoring and reporting; ② Study types: randomized controlled trials (RCTs) and non-randomized interventional studies (NRSIs); ③ Articles had to be peer-reviewed and publicly available, in either Chinese or English, and must report clear, quantifiable outcome measures;④ Language restricted to Chinese and English.

Exclusion criteria ① Studies where the intervention’s primary actor could not be clearly identified as a pharmacist, or where the research involved clinical pharmacist-led interventions but the effects could not be separated from those of general pharmacists; ② Studies that did not report explicit, extractable quantitative outcome measures, or that provided only qualitative descriptions without supporting numerical data; ③ Duplicate publications of the same study data across different journals or languages: only the version with the most complete data and the earliest publication date was retained; ④ Studies for which full text could not be obtained after reasonable attempts, or where data were missing and could not be validly retrieved through calculation or by contacting the authors; ⑤ Non-original research articles such as reviews, commentaries, expert opinions, conference abstracts, case reports, as well as purely theoretical discussions or policy interpretation pieces.

Interventions experimental group: On top of routine work, pharmacists proactively delivered pharmaceutical care with clear goal orientation and systematic planning. Their intervention behaviors showed clear features of transitioning from a traditional dispensing role toward a clinical service role.

Control group: Pharmacists continued with the traditional work model, providing only routine pharmaceutical care centered on drug supply, without implementing systematic, consciously designed pharmaceutical interventions.

Outcome measures: The outcome measures in this study fell into the following categories:

(1) Antimicrobial use indicators: changes in the pattern of antimicrobial combination therapy before and after intervention, comparison of antimicrobial prescription rationality, and improvements in various types of irrational antimicrobial use.
(2) Irrational prescriptions in intravenous admixture service centers: changes in the number and proportion of various irrational prescriptions identified through prescription review in such centers before and after pharmacist intervention.
(3) Outpatient prescription quality classification: based on relevant prescription comment management guidelines, outpatient irrational prescriptions were further classified into three types: non-standard prescriptions, inappropriate prescriptions, and exceptional prescriptions. We evaluated changes in the incidence of each type before and after intervention.

Standardization of outcome definitions.

The included studies used varying definitions for prescription rationality categories. To enhance transparency, we predefined the following operational definitions based on the national regulatory standards commonly referenced in Chinese studies:

Rational prescription: A prescription that fully complies with all relevant regulations. No violations of indication, drug selection, dosage, route, frequency, duration, or combination therapy are present.

Basically rational prescription: A prescription that meets most rationality criteria but contains minor issues that do not significantly affect clinical medication safety or efficacy (e.g., non-standard prescription writing without clinical impact).

Irrational prescription: A prescription that violates one or more of the above criteria, including inappropriate drug selection, incorrect dosage, improper route or frequency, unnecessary combination, overly long duration, or medication without indication.

Exceptional prescription: A prescription involving off-label use (e.g., unapproved indication, dosage, or patient population) without sufficient evidence or regulatory justification.

### 2.3 Literature screening and data extraction

Two researchers independently performed literature screening, data extraction, and risk of bias assessment. Their independent decisions were compared; disagreements were rare (e.g., less than 2% of records at each stage) and were resolved through discussion between the two reviewers. The pre-planned contingency of consulting a third reviewer was never needed because consensus was always reached by discussion alone. Cohen‘s kappa values were calculated to quantify inter-reviewer agreement, and they confirmed almost perfect to substantial agreement. Nevertheless, a resolution protocol was predefined: in the hypothetical event of disagreement, the two reviewers would first discuss; if no agreement could be reached, a third reviewer would make the final decision. Inter-reviewer agreement was assessed using Cohen‘s kappa, which confirmed almost perfect agreement, further supporting the absence of unresolved disagreements.

For data extraction, we used a pre-designed data extraction form. The extracted information covered the following items: study title, first author’s name, publication year, sample size, details of the intervention, and the reported outcome measures.

Identification and exclusion of overlapping populations. Because several included studies shared similar topics, settings, and intervention periods, we performed a systematic assessment to detect and exclude potentially overlapping or duplicate datasets. For studies with the same first author or same institution, we compared: (a) study periods, (b) sample sizes, (c) reported baseline proportions, and (d) hospital department names. When two studies from the same institution had overlapping time periods and highly similar sample characteristics, we contacted the corresponding authors to confirm dataset independence. In three such cases, authors confirmed that the studies were conducted in different time windows or different clinical departments, with no overlapping prescriptions. No definitive duplicate datasets were identified among the 32 included studies. However, because most studies reported aggregated prescription-level data rather than individual patient identifiers, a small degree of undetected overlap cannot be entirely ruled out.

Inter-reviewer agreement was assessed using Cohen‘s kappa statistic (with 95% confidence intervals) for both the title/abstract screening and full-text screening stages, based on the independent decisions of the two reviewers before any consensus discussion. Any disagreements that arose were resolved through discussion between the two reviewers; a third reviewer was not required.

### 2.4 Literature quality assessment

For the included RCTs, we used the Cochrane risk of bias (ROB) tool. This tool covers six domains: selection bias, performance bias, detection bias, attrition bias, reporting bias, and other bias. It allows a comprehensive evaluation of a study’s risk of bias.

For the included NRSIs, we used the ROBINS-I tool (Risk Of Bias In Non-randomized Studies of Interventions). This tool includes seven bias domains: confounding, selection of participants, classification of interventions, deviations from intended interventions, missing data, measurement of outcomes, and selective reporting. By answering 33 signaling questions across these domains, one can judge the risk of bias for each domain and then determine the overall risk of bias for the study. The detailed domain-level judgments for each included NRSI, including the supporting justifications based on the 33 signaling questions of the ROBINS-I tool, are provided in Supplementary Table S7. This table allows readers to examine the basis for each risk-of-bias rating. The signaling question responses are available from the corresponding author upon request.

Inter-reviewer agreement for the risk of bias assessments (using Cochrane ROB tool for RCTs and ROBINS-I for NRSIs) was also evaluated using Cohen‘s kappa (*κ* = 0.74, substantial agreement). Occasional disagreements were resolved by discussion; no third-party arbitration was needed.

### 2.5 Statistical analysis

We entered and initially managed the data using Microsoft Excel. All meta-analytical calculations were performed with RevMan 5.4. For count data, we used the odds ratio (OR) with its 95% confidence interval (CI) as the effect measure.

Data extraction from NRSIs. For non-randomized interventional studies (NRSIs), we extracted unadjusted (crude) count data (e.g., number of rational/irrational prescriptions before and after intervention). Most NRSIs employed a pre-post self-controlled design and did not report adjusted effect estimates. When studies reported both crude and adjusted estimates (e.g., logistic regression results), we used the crude data to maintain consistency across studies. We acknowledge that unadjusted estimates from NRSIs are subject to confounding and may overestimate intervention effects; this limitation is addressed in the Discussion.

Heterogeneity across studies was assessed quantitatively using the *χ*^2^ test, together with the *I*^2^ statistic to gauge the degree of heterogeneity. When studies showed good homogeneity (*I*^2^ < 50%), we applied a fixed-effect model to pool the effect sizes. When heterogeneity was substantial (*I*^2^ ≥ 50%), we switched to a random-effects model to account for the impact of between-study variation on the pooled estimate (9, 10).

#### 2.5.1 Justification for pooling RCTs and NRSIs

This review included both randomized controlled trials (RCTs; *n* = 5) and non-randomized interventional studies (NRSIs; *n* = 27). Pooling RCTs and NRSIs is methodologically debatable, as non-randomized studies are inherently more susceptible to bias and confounding. However, given the very small number of RCTs (only 5) and the exploratory nature of this meta-analysis on an emerging topic (pharmacist role transformation in China), we provisionally pooled all eligible studies to obtain overall effect estimates, with the explicit understanding that these findings should be considered hypothesis-generating. A random-effects model was used for all pooled analyses to account for anticipated clinical and methodological heterogeneity, including differences in study design. To critically assess whether pooling RCTs and NRSIs distorted the results, we conducted subgroup analyses stratified by study design (RCT vs. NRSI) for the three core outcomes. In addition, sensitivity analyses excluding high-risk NRSIs were performed. The effect directions and magnitudes were consistent across RCTs and NRSIs, and the results remained stable after excluding high-risk studies. These analyses support the robustness of the combined estimates, but the findings should be interpreted with caution given the methodological debate.

To evaluate potential publication bias among the included studies, we first constructed funnel plots for the core outcomes with at least 10 included studies (e.g., single-agent antimicrobial use, rational prescriptions, and irrational prescriptions) to obtain a preliminary visual assessment. We then performed Egger’s linear regression test using Stata 14.0 to provide a quantitative evaluation of funnel plot asymmetry (11).

### 2.6 Certainty of evidence assessment (GRADE)

The certainty of evidence for the three core outcomes (single-agent antimicrobial use, rational antimicrobial prescriptions, and irrational antimicrobial prescriptions) was assessed using the GRADE (Grading of Recommendations Assessment, Development and Evaluation) approach (12). Evidence was rated as high, moderate, low, or very low based on five domains: risk of bias, inconsistency, indirectness, imprecision, and publication bias. Risk of bias was derived from the Cochrane ROB tool for RCTs and ROBINS-I for NRSIs. Inconsistency was evaluated using *I*^2^ statistics and the overlap of 95% confidence intervals. Indirectness considered whether outcomes were process-based rather than patient-centered. Imprecision was judged based on the optimal information size (OIS) and the width of confidence intervals. Publication bias was assessed using Egger‘s tests and trim-and-fill analysis (13). The GRADE assessment was performed using GRADEpro GDT software (McMaster University, 2020). The results are summarized in Supplementary Table S6.

## 3 Results

### 3.1 Literature screening process

**Figure 1:**
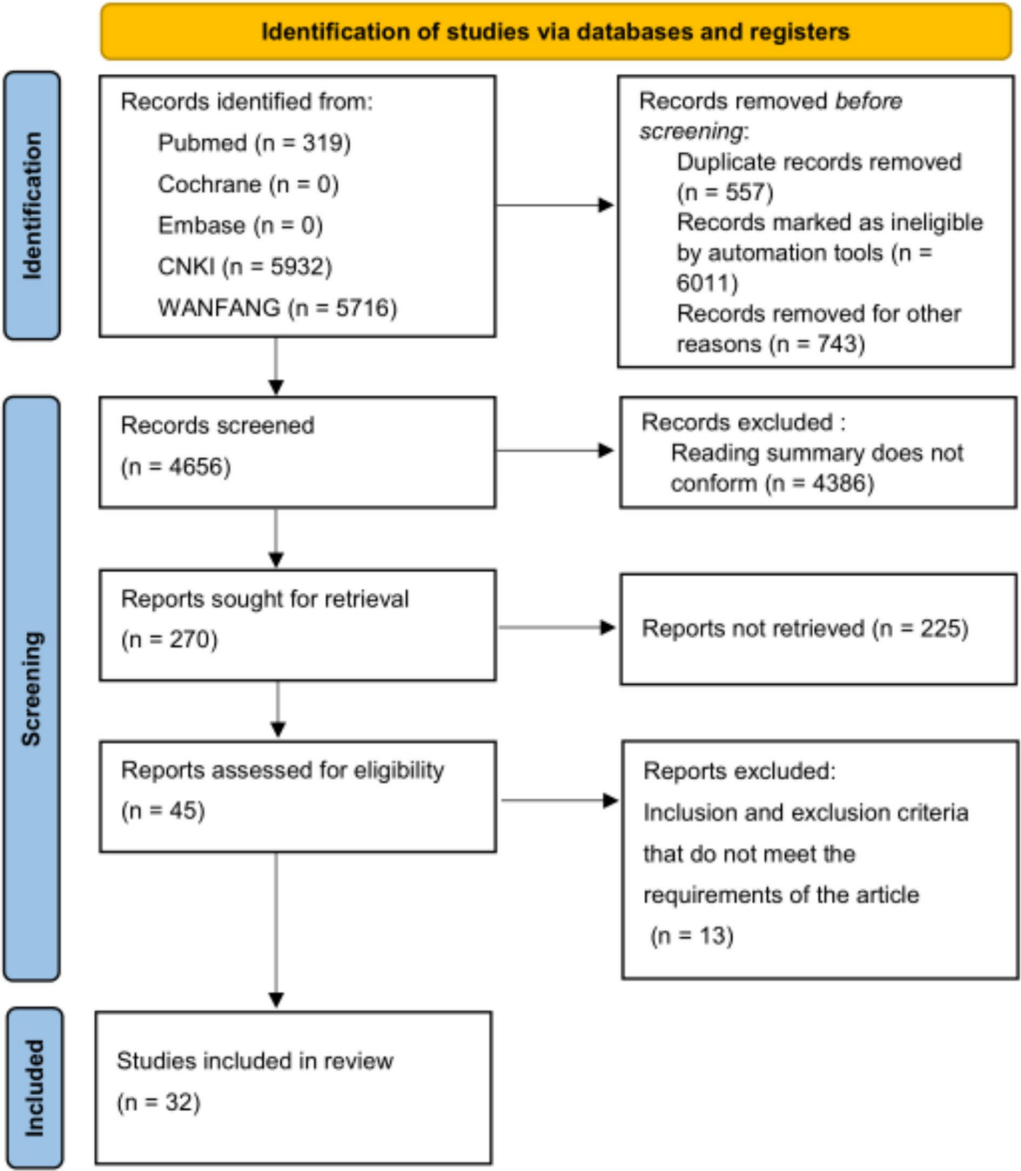
Flow diagram of the selection process of literature.

Our initial search retrieved 11,648 Chinese records and 319 English records. After removing duplicates, screening titles and abstracts, and reading full texts, we finally included 32 studies (14–45), all of which were in Chinese. Among them, five were RCTs (19, 23, 32, 41, 45) and 27 were NRSIs (13–18, 20–22, 24–31, 33–40, 42–44). The study selection process is shown in Figure 1.

### 3.2 Basic characteristics of the included literature

Among the 32 included studies, 27 examined pharmaceutical care delivered by pharmacists in outpatient pharmacies. Of these, roughly 20 focused primarily on the impact of pharmacist interventions on antimicrobial prescriptions, specifically changes in antimicrobial use rationality, combination patterns (single-agent, two-drug, and three-or-more-drug combinations), and various forms of irrational use (e.g., overly long treatment duration, inappropriate drug selection, incorrect dosage or administration, and medication without indication). The remaining outpatient pharmacy studies involved pharmacists conducting prescription review, medication instruction, pharmacy training, and interventions on combined Chinese-Western medicine use, with outcome measures including prescription qualification rates, patient medication adherence, satisfaction levels, and adverse drug reaction incidence.

Another five studies examined pharmaceutical care delivered by pharmacists in pharmacy intravenous admixture service centers (PIVAS). Interventions in these studies mainly included pharmacist review of physician orders, standardization of the prescription review process, enhanced communication between pharmacists and medical/nursing staff, training, and administrative interventions. Outcome measures included types of irrational orders (e.g., inappropriate solvent selection, improper route/sequence/dosage), order review rates, incidence of medication-related adverse events, and management quality scores.

No studies in this set explicitly used inpatient pharmacies as an independent study setting. The basic characteristics of the included studies are presented in Tables 1, 2, and 3.

**Table 1:** Characteristics of antibiotic intervention studies.

Author	Year	Group	No.	Comparison of combined use of antibiotics			Comparison of rationality of drugs			Comparison of unreasonable situation of antibacterial drugs						
				Single	Dual	Triple	Reasonable prescription	Basically reasonable prescription	Unreasonable prescription	Overly long treatment duration	Inappropriate drug selection	Incorrect dosage/administration	Inappropriate timing of administration	Unnecessary combination therapy	Failure to prescribe by generic name	Medication without indication
Chen (14)	2018	Pre-intervention‌	120	29	73	18	69	14	37	74	26	65	37	73	65	71
		Intervention	120	69	50	1	103	2	15	9	14	26	8	53	0	35
Zong (15)	2018	Pre-intervention‌	100	30	63	7	58	11	31	–	–	–	–	–	–	–
		Intervention	100	61	38	1	82	7	11	–	–	–	–	–	–	–
Zhou (16)	2019	Pre-intervention‌	35	15	12	8	16	12	7	–	–	–	–	–	–	–
		Intervention	35	18	15	2	19	15	1	–	–	–	–	–	–	–
Gao (17)	2019	Pre-intervention‌	350	165	145	54	189	126	35	10	6	6	–	–	–	–
		Intervention	350	195	101	40	165	173	12	4	3	3	–	–	–	–
Hu (18)	2020	Pre-intervention‌	34	–	–	–	–	–	–	–	3	3	4	3	–	–
		Intervention	34	–	–	–	–	–	–	–	1	0	1	1	–	–
Guo (19)	2018	Pre-intervention‌	600	164	396	40	347	64	189	–	450	265	–	–	320	371
		Intervention	600	348	246	6	510	50	40	–	46	150	–	–	0	254
Wu (20)	2018	Pre-intervention‌	30	7	20	3	18	5	7	–	–	–	–	–	–	–
		Intervention	30	18	11	1	25	4	1	–	–	–	–	–	–	–
Liao (21)	2019	Pre-intervention‌	300	158	110	32	178	40	62	–	–	–	–	–	–	–
		Intervention	300	184	100	16	244	38	18	–	–	–	–	–	–	–
Zhang (22)	2015	Pre-intervention‌	500	136	334	30	289	53	158	119	32	80	46	110	86	98
		Intervention	500	290	205	5	404	30	66	5	11	30	10	32	0	34
Wang (23)	2022	Pre-intervention‌	150	75	54	21	–	–	–	–	–	4	3	–	–	–
		Intervention	150	124	24	2	–	–	–	–	–	1	0	–	–	–
Deng (24)	2019	Pre-intervention‌	1,000	–	–	–	–	–	–	60	62	82	–	101	–	80
		Intervention	1,000	–	–	–	–	–	–	15	10	27	–	28	–	20
Wang (25)	2019	Pre-intervention‌	100	32	50	18	–	–	–	–	–	–	–	–	–	–
		Intervention	100	61	31	8	–	–	–	–	–	–	–	–	–	–
Geng (26)	2019	Pre-intervention‌	500	138	332	30	287	52	161	–	370	225	–	–	264	315
		Intervention	500	285	210	5	425	39	36	–	35	120	–	–	0	213
Jia (27)	2019	Pre-intervention‌	40	14	24	2	15	18	7	–	–	–	–	–	–	–
		Intervention	40	23	16	1	24	15	1	–	–	–	–	–	–	–
Chen (28)	2019	Pre-intervention‌	38	20	12	6	–	–	–	6	4	6	–	6	–	4
		Intervention	38	33	4	1	–	–	–	1	0	1	–	1	–	0
Yang (29)	2018	Pre-intervention‌	148	68	65	15	88	41	19	6	4	3	–	–	–	–
		Intervention	148	94	48	6	123	19	6	2	1	1	–	–	–	–
Zou (30)	2020	Pre-intervention‌	300	89	132	19	–	–	–	–	8	10	–	11	–	7
		Intervention	300	196	80	4	–	–	–	–	2	2	–	1	–	1
Zhuo (31)	2023	Pre-intervention‌	100	51	32	17	35	52	13	–	–	–	–	–	–	–
		Intervention	100	79	16	5	57	41	2	–	–	–	–	–	–	–
Tang (32)	2019	Pre-intervention‌	80	20	37	23	39	26	15	–	–	–	–	–	–	–
		Intervention	80	53	21	6	45	33	2	–	–	–	–	–	–	–
Geng (33)	2019	Pre-intervention‌	50	–	–	–	8	14	28	–	–	–	–	–	–	–
		Intervention	50	–	–	–	28	22	0	–	–	–	–	–	–	–
Yao (34)	2019	Pre-intervention‌	50	15	23	12	20	20	10	–	–	–	–	–	–	–
		Intervention	50	28	21	1	40	9	1	–	–	–	–	–	–	–

–, Data not provided.

**Table 2:** Characteristics of antibiotic intervention studies.

Author	Year	Group	No.	Comparison of unreasonable prescriptions		
				Irrational drug dosage	Unreasonable choice of solvent	Unreasonable route of administration
Cai (35)	2025	Pre-intervention‌	500	32	19	9
		Intervention	500	7	3	1
Gu (36)	2019	Pre-intervention‌	110	10	9	–
		Intervention	110	3	1	–
Che (37)	2021	Pre-intervention‌	10,000	543	156	74
		Intervention	10,000	74	32	25
Huang (38)	2024	Pre-intervention‌	11,000	–	95	100
		Intervention	12,500	–	48	36
Zhang (39)	2021	Pre-intervention‌	12,720	35	35	10
		Intervention	13,680	4	7	3

–, Data not provided.

**Table 3:** Characteristics of antibiotic intervention studies.

Author	Year	Group	No.	Non-standard prescription			Inappropriate prescription							Exceptional prescription
				Incomplete diagnostic writing or missing prescription content	Prescription writing, physician signature is not standardized, modify the unsigned	No special circumstances, outpatient prescription > 7 d dosage, emergency prescription > 3 d dosage	Drug selection is inappropriate	Indications not suitable	The dosage form of is not suitable	Administration route is not suitable	Usage and dosage are inappropriate	Drug combination is not suitable	Repeated medication	Exceptional prescription
Zhu (40)	2021	Pre-intervention‌	156,353	1,852	408	248	1,712	4,036	686	552	3,744	3,008	1,588	928
		Intervention	165,676	831	253	145	435	2,062	642	557	1,344	1,508	392	115
Yang (41)	2020	Pre-intervention‌	6,000	23	18	3	23	15	31	313	23	25	20	2
		Intervention	6,000	10	6	2	22	10	14	154	26	15	11	2
Zhu (42)	2018	Pre-intervention‌	600	–	–	–	–	–	–	–	–	11	21	–
		Intervention	600	–	–	–	–	–	–	–	–	10	12	–
Liu (43)	2022	Pre-intervention‌	1,000	41	33	2	8	24	11	8	19	9	25	2
		Intervention	1,000	21	12	1	7	10	5	3	9	5	16	1
Yang (44)	2020	Pre-intervention‌	1,206	45	14	20	–	13	–	11	35	–	1	4
		Intervention	1,084	19	5	7	–	4	–	3	12	–	1	1
Zhong (45)	2019	Pre-intervention‌	40	–	–	–	–	–	–	–	5	2	1	–
		Intervention	40	–	–	–	–	–	–	–	1	1	0	–

–, Data not provided.

### 3.3 Quality assessment

**Figure 2:**
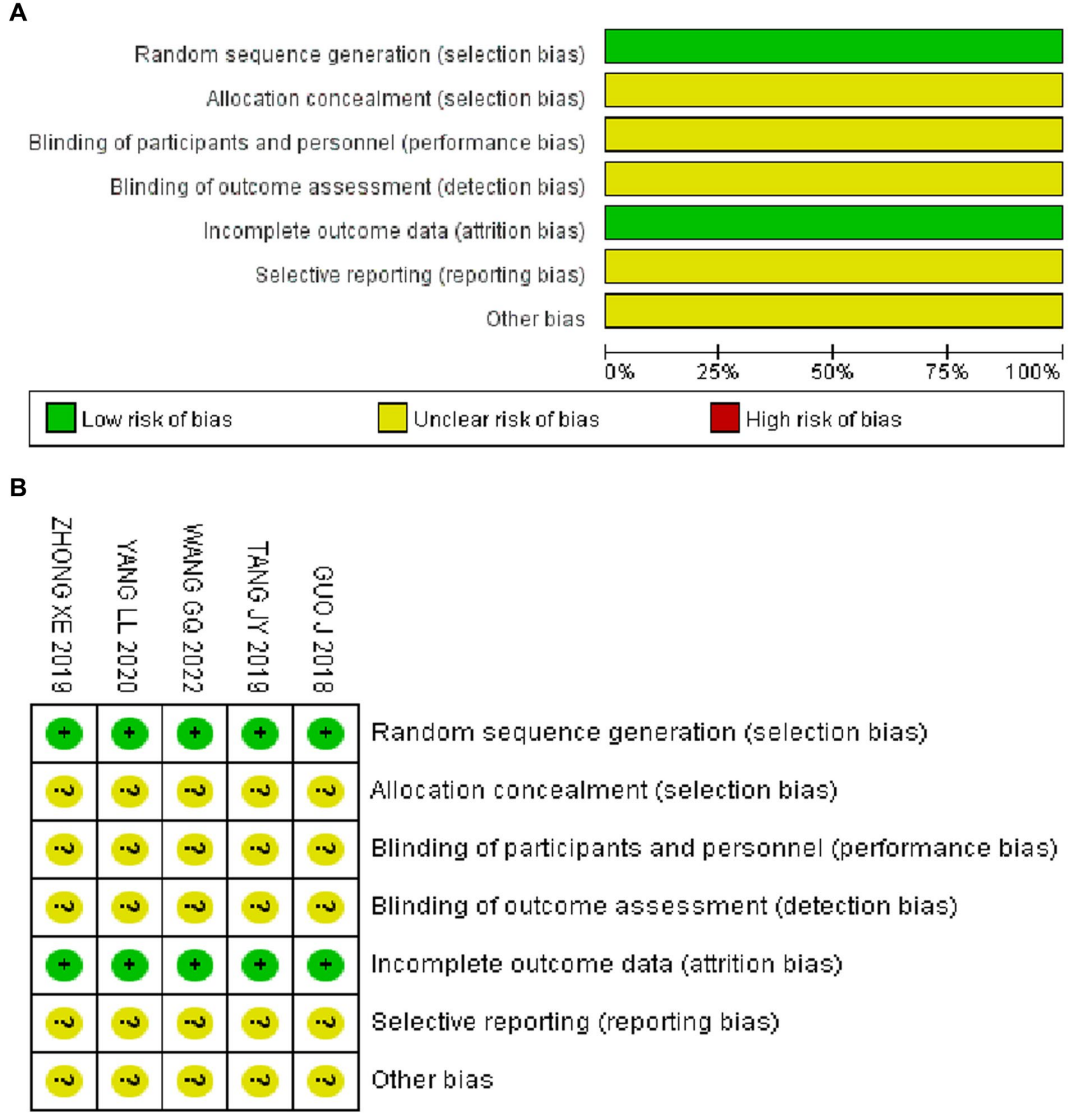
**(A)** Bar chart of bias risk. **(B)** Bias risk map.

For the five included RCTs (19, 23, 32, 41, 45), we used the ROB tool. In the selection bias domain, allocation concealment, along with performance bias, detection bias, reporting bias, and other bias, were generally judged as having unclear risk due to missing information. For random sequence generation (also within the selection bias domain), all five studies described their randomization methods, leading to a judgment of low risk. Attrition bias was rated as low risk across all five studies. The distribution of biases is shown in Figures 2A,B.

For the 27 included NRSIs (13–18, 20–22, 24–31, 33–40, 42–44), we applied the ROBINS-I tool. Of these, 23 were judged as having moderate risk of bias, and four as high risk. Across the seven bias domains, five domains – selection of participants, classification of interventions, deviations from intended interventions, missing data, and selective reporting – were mostly rated as low risk. For confounding bias, several studies did not describe whether the baseline characteristics of the experimental and control groups were comparable, leaving potential confounding factors that could influence the intervention effect; these were rated as high risk. For the measurement of outcomes domain, because we could not determine whether outcome assessors were aware of the intervention received by participants, all studies received a moderate risk rating. The results are presented in Table 4.

**Table 4:** Bias assessment of enrolled NRSIs.

Author	Selection of subject bias	Intervention classification bias	Intentional intervention deviation bias	Lost data bias	Selective reporting bias	Overall risk
Chen (14)	Moderate	Low	Moderate	Low	Low	Moderate
Zong (15)	Moderate	Low	Moderate	Low	Low	Moderate
Zhou (16)	Moderate	Low	Low	Low	Low	Moderate
Gao (17)	Moderate	Low	Moderate	Low	Low	Moderate
Hu (18)	Moderate	Low	Moderate	Low	Low	Moderate
Wu (20)	Moderate	Low	Moderate	Low	Low	Moderate
Liao (21)	Moderate	Low	Moderate	Low	Low	Moderate
Zhang (22)	High	Low	High	Moderate	Low	High
Deng (24)	Moderate	Low	Moderate	Low	Low	Moderate
Wang (25)	Moderate	Low	Moderate	Low	Low	Moderate
Geng (26)	Moderate	Low	Moderate	Low	Low	Moderate
Jia (27)	Moderate	Low	Moderate	Low	Low	Moderate
Chen (28)	Moderate	Low	Moderate	Low	Low	Moderate
Yang (29)	Moderate	Low	Moderate	Low	Low	Moderate
Zou (30)	Moderate	Low	Moderate	Low	Low	Moderate
Zuo (31)	Moderate	Low	Low	Low	Low	Moderate
Geng (33)	Moderate	Low	Moderate	Moderate	Low	Moderate
Yao (34)	Moderate	Low	Moderate	Low	Low	Moderate
Cai (35)	Moderate	Low	Moderate	Low	Low	Moderate
Gu (36)	Moderate	Low	Moderate	Moderate	Low	Moderate
Che (37)	High	Low	High	High	Low	High
Huagn (38)	High	Low	High	Moderate	Low	High
Zhagn (39)	High	Low	High	Moderate	Low	High
Zhu (40)	Moderate	Low	Moderate	Low	Low	Moderate
Zhu (42)	Moderate	Low	Moderate	Low	Low	Moderate
Liu (43)	Moderate	Low	Moderate	Low	Low	Moderate
Yang (44)	Moderate	Low	Moderate	Low	Low	Moderate

The domain-level assessments (Supplementary Table S7) revealed that confounding was the most frequent concern: 4 studies were rated high risk due to lack of baseline comparability, and the remaining NRSIs were rated moderate risk because they did not report adjusted estimates. Deviations from intended interventions were rated high risk in the same 4 studies, as blinding was not possible and co-interventions were not controlled. Measurement of outcomes was uniformly rated moderate because outcome assessors were not explicitly blinded. These judgments directly guided our analytical decisions: (i) the 4 high-risk studies were excluded in a sensitivity analysis (Results 3.9) to assess their impact; (ii) the GRADE assessment (Results 3.11) downgraded certainty of evidence for risk of bias due to the predominance of moderate- and high-risk NRSIs; and (iii) the Discussion highlights that residual confounding cannot be excluded, and therefore the pooled estimates should be interpreted with caution.

The inter-reviewer agreement for title/abstract screening was almost perfect (*κ* = 0.87, 95%CI 0.84–0.90), for full-text screening was substantial (*κ* = 0.79, 95%CI 0.72–0.86), and for risk of bias assessments was also substantial (*κ* = 0.74, 95%CI 0.65–0.83).

### 3.4 Impact of pharmacist interventions on antimicrobial prescriptions

#### 3.4.1 Antimicrobial combination patterns before and after pharmacist intervention

Based on the forest plot results, the meta-analysis of antimicrobial combination patterns before and after pharmacist intervention included 18 studies with 7,082 prescriptions, divided into three subgroups: single-agent, two-drug, and three-or-more-drug combinations.

For the single-agent subgroup, the pooled result showed that the proportion of single-agent antimicrobial use after pharmacist intervention was significantly higher than before intervention [OR = 3.13, 95%CI (2.49, 3.92), *p* < 0.00001], with substantial heterogeneity (*I*^2^ = 77%). Finding suggests that pharmacist intervention may increase the rate of single-agent antimicrobial use and reduce unnecessary combination therapy, aligning with clinical expectations. However, the substantial heterogeneity (*I*^2^ = 77%) limits the precision of this estimate, and the exact effect size should be interpreted cautiously.

For the two-drug and three-or-more-drug subgroups, the pooled results also indicated significant reductions after intervention. The proportion of two-drug combinations dropped [OR = 0.46, 95%CI (0.39, 0.55), *p* < 0.00001], as did the proportion of three-or-more-drug combinations [OR = 0.24, 95%CI (0.16, 0.35), *p* < 0.00001]. Heterogeneity was moderate for both subgroups (*I*^2^ = 57 and 52%, respectively), and we applied a random-effects model. These findings are consistent with those of the single-agent subgroup, reinforcing that pharmacist interventions effectively reduce combination therapy. The results are presented in Table 5.

**Table 5:** Meta-analysis results of pharmacists intervene in antibiotics.

Outcomes	Studies	Participants	Heterogeneity test results		Effect model	Meta-analysis results	
			*p*	*I* ^2^		95%CI	*p*
Comparison of combined use of antibiotics							
Single	18	7,082	<0.00001	77%	Random	3.13[2.49, 3.92]	<0.00001
Dual	18	7,082	0.001	57%	Random	0.46[0.39, 0.55]	<0.00001
Triple	18	7,082	0.006	52%	Random	0.24[0.16, 0.35]	<0.00001
Comparison of rationality of drug use							
Reasonable prescription	15	6,006	<0.00001	75%	Random	2.97[2.30, 3.85]	<0.00001
Basically reasonable prescription	15	6,006	0.009	52%	Random	0.70[0.56, 0.89]	0.003
Unreasonable prescription	15	6,006	0.05	41%	Fixed	0.22[0.17, 0.28]	<0.00001
Comparison of unreasonable situation of antibacterial drugs							
Overly long treatment duration	6	4,312	0.0002	80%	Random	0.13[0.05, 0.33]	<0.0001
Inappropriate drug selection	10	7,180	<0.00001	92%	Random	0.15[0.06, 0.38]	<0.0001
Incorrect dosage/administration	11	7,480	0.67	0%	Fixed	0.36[0.31, 0.42]	<0.00001
Inappropriate timing of administration	4	1,608	0.97	0%	Fixed	0.18[0.11, 0.30]	<0.00001
Unnecessary combination therapy	5	3,908	0.14	43%	Fixed	0.30[0.20, 0.44]	<0.00001
Failure to prescribe by generic name	4	3,440	0.70	0%	Fixed	0.00[0.00, 0.01]	<0.00001
Medication without indication	7	6,116	0.09	46%	Fixed	0.35[0.28, 0.44]	<0.00001

All three subgroups showed moderate to substantial heterogeneity (*I*^2^ = 77, 57, and 52%, respectively). Several factors likely contributed to this heterogeneity: differences in intervention types across studies (e.g., simple prescription review versus comprehensive interventions, prospective interception versus retrospective comment, different hospital levels and specialty types, outpatient versus PIVAS settings); variations in study design (e.g., pre-post comparisons within the same group versus historical controls), along with some studies carrying a high risk of bias (four studies rated as high risk); wide variation in sample sizes (ranging from 40 prescriptions to over 160,000); and random error due to small event counts in some studies within the three-drug subgroup. Together, this clinical and methodological diversity contributed to the statistical heterogeneity observed in the pooled effect estimates. Given this heterogeneity, the precise pooled ORs should not be overinterpreted; rather, the consistent direction of effect across studies is the more reliable finding.

#### 3.4.2 Comparison of rational antimicrobial prescriptions before and after pharmacist intervention

The meta-analysis comparing antimicrobial prescription rationality before and after pharmacist intervention included 15 studies (the same number of studies in each subgroup), with a total sample size of approximately 6,006 prescriptions.

For the rational prescription subgroup, the pooled result showed that the proportion of rational prescriptions after pharmacist intervention was significantly higher than before intervention [OR = 2.97, 95%CI (2.30, 3.85), *p* < 0.00001], with substantial heterogeneity (*I*^2^ = 75%). For the basically rational prescription subgroup, the pooled OR was 0.70, 95%CI (0.56, 0.89), *p* = 0.003, with moderate heterogeneity (*I*^2^ = 52%). For the irrational prescription subgroup, the pooled OR was 0.22, 95%CI (0.17, 0.28), *p* < 0.00001, with moderate heterogeneity (*I*^2^ = 41%). These findings indicate that pharmacist intervention is associated with an increase in the rate of rational antimicrobial prescriptions and reductions in basically rational and irrational prescriptions. Nevertheless, the substantial heterogeneity (*I*^2^ = 75% for rational prescriptions) cautions against overreliance on the exact OR values. Results are presented in Table 5.

All three subgroups showed some degree of heterogeneity (rational prescriptions *I*^2^ = 75%, basically rational *I*^2^ = 52%, irrational *I*^2^ = 41%). Sources of heterogeneity likely include: inconsistent criteria across studies for defining “rational,” “basically rational,” and “irrational” (e.g., some studies referred to the Guiding Principles for Clinical Application of Antimicrobial Agents, others to drug package inserts or hospital-specific standards); large variations in interventions (from simple prescription review to combined training plus administrative intervention); different study settings (outpatient pharmacies versus PIVAS centers, different hospital levels); and relatively small sample sizes in some studies, which can cause large fluctuations in effect estimates and further increase statistical heterogeneity.

#### 3.4.3 Comparison of specific types of irrational antimicrobial use before and after pharmacist intervention

The meta-analysis results indicated that all examined types of irrational antimicrobial use improved significantly after pharmacist intervention. For each type, the pooled effect size was below 1, and all *p* values were less than 0.0001: overly long treatment duration [OR = 0.13, 95%CI (0.05, 0.33)], inappropriate drug selection [OR = 0.15, 95%CI (0.06, 0.38)], incorrect dosage [OR = 0.36, 95%CI (0.31, 0.42)], improper timing of administration [OR = 0.18, 95%CI (0.11, 0.30)], unnecessary combination therapy [OR = 0.30, 95%CI (0.20, 0.44)], failure to use generic names for antimicrobials [OR = 0.00, 95%CI (0.00, 0.01)], and antimicrobial use without indication [OR = 0.35, 95%CI (0.28, 0.44)]. These findings demonstrate that pharmacist intervention effectively reduces the incidence of the above irrational prescribing practices.

Regarding heterogeneity, the *I*^2^ values for “incorrect dosage,” “improper timing of administration”, and “failure to use generic names” were all 0%, so we used a fixed-effect model. “Unnecessary combination therapy” (*I*^2^ = 43%) and “use without indication” (*I*^2^ = 46%) had low heterogeneity, also analyzed with a fixed-effect model. “Overly long treatment duration” (*I*^2^ = 80%) and “inappropriate drug selection” (*I*^2^ = 92%) showed substantial heterogeneity, leading us to use a random-effects model; this likely relates to differences across studies in intervention intensity, baseline rates of irrational use, and prescription sources. Overall, pharmacist interventions appear to be associated with reduced irrational antimicrobial use, although the substantial heterogeneity for some outcomes (e.g., inappropriate drug selection, *I*^2^ = 92%) indicates that the precise effect sizes are uncertain. Results are shown in Table 5.

### 3.5 Impact of pharmacist interventions on irrational prescriptions in intravenous admixture service centers

The analysis of pharmacist interventions on irrational prescriptions in intravenous admixture service centers showed that after intervention, the proportion of irrational prescriptions decreased significantly. The pooled effect size was OR = 0.22, 95%CI (0.16, 0.31), *p* < 0.00001, with heterogeneity *I*^2^ = 73%; we therefore applied a random-effects model for the overall analysis.

For the subgroup of incorrect drug dosage, the pooled OR was 0.14, 95%CI (0.11, 0.17), *p* < 0.00001, with *I*^2^ = 0%. For the subgroup of inappropriate solvent selection, the pooled OR was 0.24, 95%CI (0.14, 0.40), *p* < 0.00001, with moderate heterogeneity (*I*^2^ = 68%). For the subgroup of improper route of administration, the pooled OR was 0.31, 95%CI (0.24, 0.42), *p* < 0.00001, with *I*^2^ = 0%. The pooled effect sizes for all three subgroups were significantly below 1, indicating that pharmacist intervention effectively reduces the incidence of incorrect dosage, inappropriate solvent selection, and improper administration route in intravenous admixture service center prescriptions.

**Figure 3:**
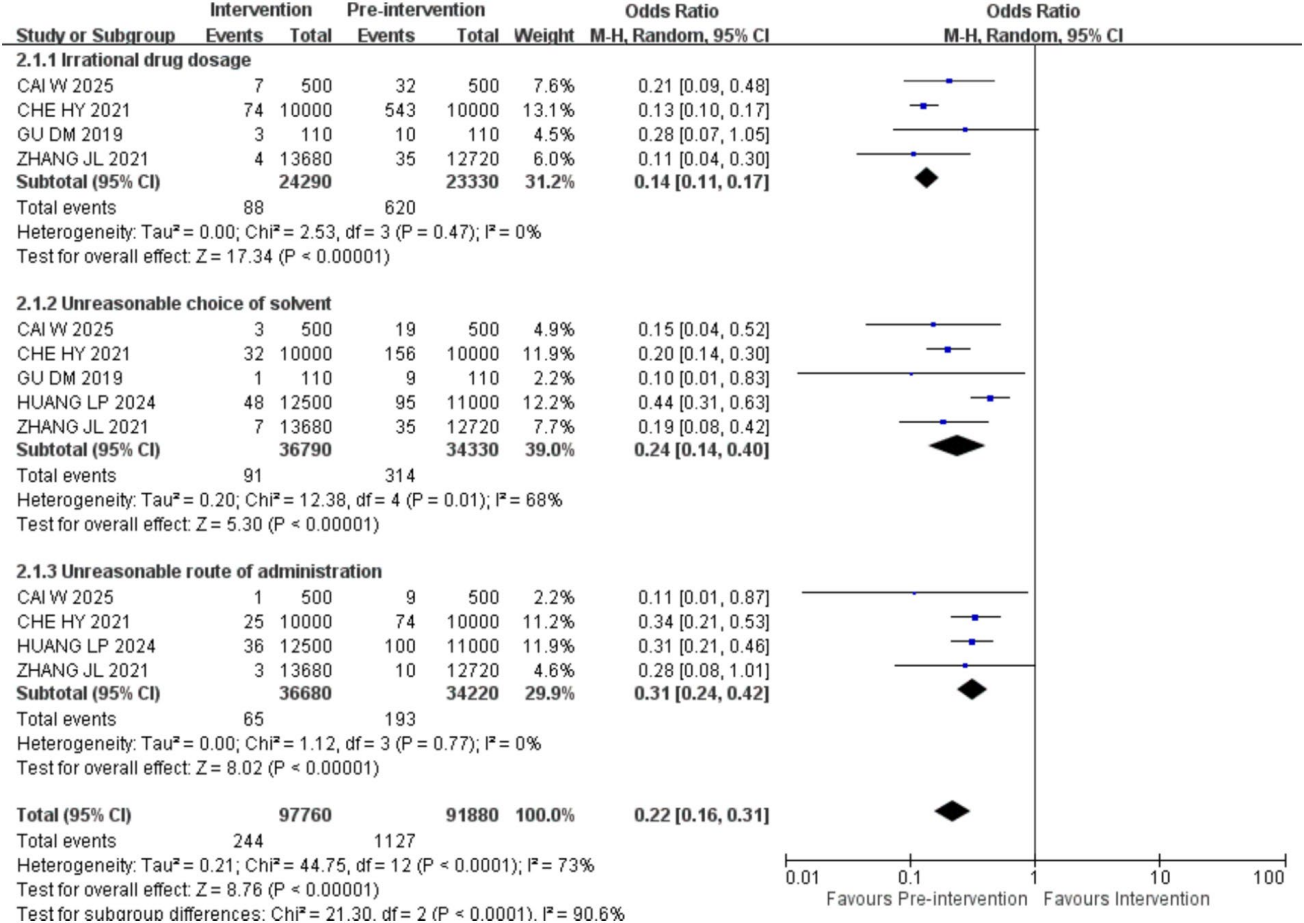
Meta-analysis results of Comparison of unreasonable prescriptions.

The test for subgroup differences was statistically significant (Chi^2^ = 21.30, *p* < 0.00001, *I*^2^ = 90.6%), suggesting that different types of irrational prescribing respond differently to the intervention. The overall heterogeneity was substantial (*I*^2^ = 73%), mainly driven by between-study variation within the solvent selection subgroup. Therefore, while the reduction in irrational prescriptions appears consistent across studies, the pooled OR should be interpreted with caution. Results are shown in Figure 3.

### 3.6 Impact of pharmacist interventions on irrational outpatient prescriptions

#### 3.6.1 Non-standard prescriptions

The analysis of pharmacist interventions on non-standard outpatient prescriptions showed the following results. For the subgroup “incomplete diagnosis or missing prescription items”, the pooled OR was 0.42, 95%CI (0.39, 0.46), *p* < 0.00001, with *I*^2^ = 0%. For the subgroup “non-standard prescription writing, irregular physician signatures, or unsigned modifications”, the pooled OR was 0.56, 95%CI (0.48, 0.64), *p* < 0.00001, with low heterogeneity (*I*^2^ = 17%). For the subgroup “outpatient prescriptions exceeding 7 days’ supply or emergency prescriptions exceeding 3 days’ supply without special justification”, the pooled OR was 0.54, 95%CI (0.45, 0.65), *p* < 0.00001, with *I*^2^ = 0%. The pooled effect sizes for all three subgroups were significantly below 1, indicating that pharmacist intervention effectively reduces the incidence of various types of non-standard prescriptions. The most pronounced improvement was seen for incomplete diagnosis or missing items (OR = 0.42).

**Figure 4:**
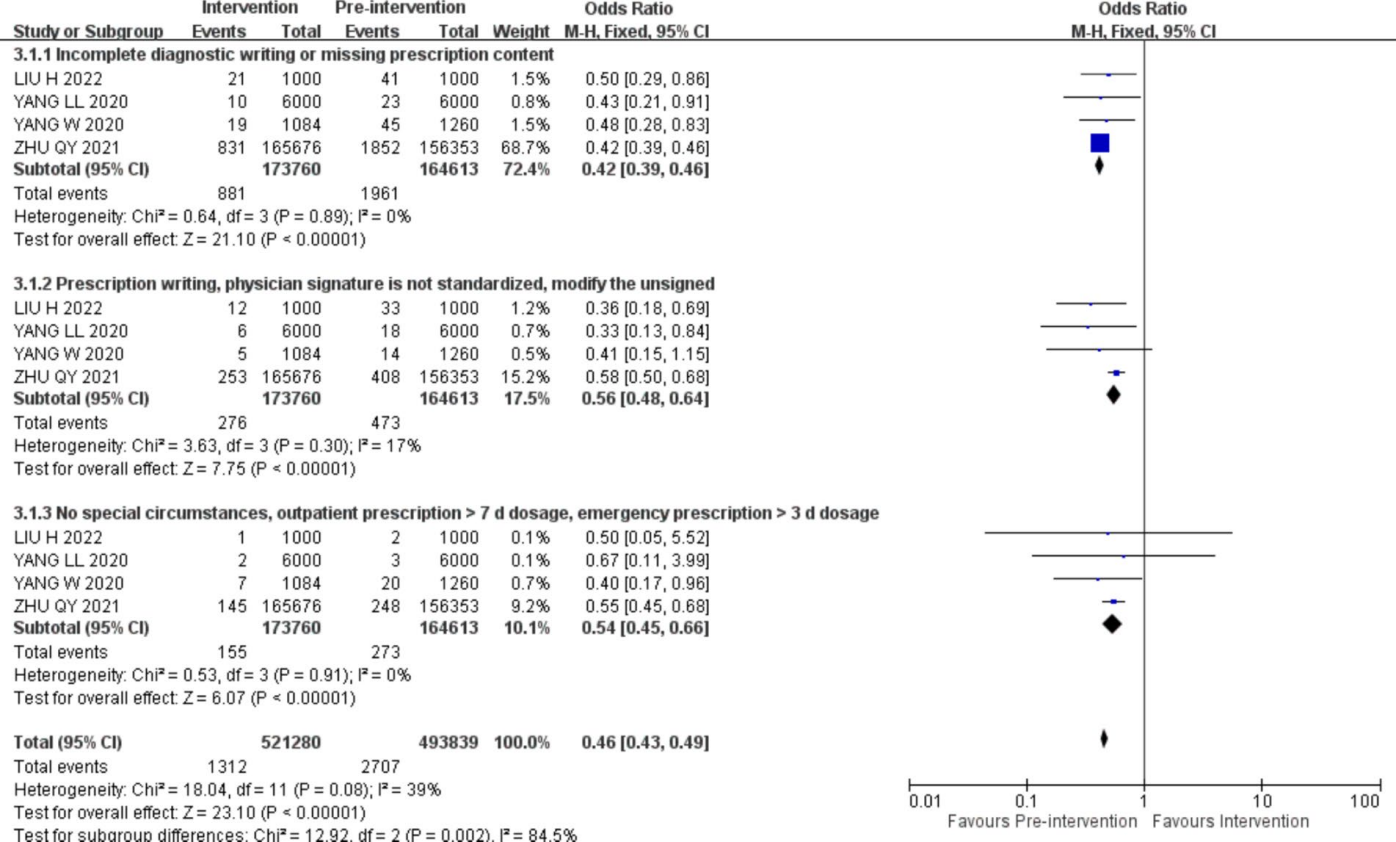
Meta-analysis results of non-standard prescription.

The overall pooled effect size was OR = 0.46, 95%CI (0.43, 0.49), *p* < 0.00001, with low heterogeneity (*I*^2^ = 39%), and we used a fixed-effect model. The difference between subgroups was not statistically significant (as the confidence intervals overlapped well), and the overall result appears robust. The heterogeneity mainly came from individual studies within the “non-standard prescription writing” subgroup (e.g., Zhu 2021 had the largest weight, with an OR of 0.58, slightly higher than other studies), but the overall heterogeneity remained within an acceptable range. In summary, pharmacist interventions significantly reduce non-standard practices in outpatient prescriptions and improve the completeness and standardization of prescription writing. Results are shown in Figure 4.

#### 3.6.2 Inappropriate prescriptions

The meta-analysis of pharmacist interventions on inappropriate outpatient prescriptions included seven subgroups.

For “inappropriate indication,” the pooled OR was 0.48, 95%CI (0.45, 0.50), *p* < 0.00001, with *I*^2^ = 0%; For “inappropriate drug combination”, the pooled OR was 0.47, 95%CI (0.44, 0.50), *p* < 0.00001, with *I*^2^ = 0%; For “inappropriate dosage or administration frequency”, the pooled OR was 0.47, 95%CI (0.27, 0.84), *p* = 0.01, with substantial heterogeneity (*I*^2^ = 79%); For “duplicate medication”, the pooled OR was 0.45, 95%CI (0.24, 0.83), p = 0.01, with substantial heterogeneity (*I*^2^ = 76%); For “inappropriate route of administration”, the pooled OR was 0.57, 95%CI (0.32, 1.00), *p* = 0.05, with high heterogeneity (*I*^2^ = 92%); For “inappropriate dosage form”, the pooled OR was 0.64, 95%CI (0.37, 1.10), *p* = 0.10, with moderate heterogeneity (*I*^2^ = 65%); For “inappropriate drug selection”, the pooled OR was 0.55, 95%CI (0.18, 1.69), *p* = 0.30, with very high heterogeneity (*I*^2^ = 93%), and the difference was not statistically significant.

Overall, pharmacist interventions significantly reduce the incidence of inappropriate indications, inappropriate drug combinations, inappropriate dosage/frequency, and duplicate medication. However, the improvements for inappropriate drug selection, inappropriate dosage form, and inappropriate route of administration did not reach statistical significance.

**Figure 5:**
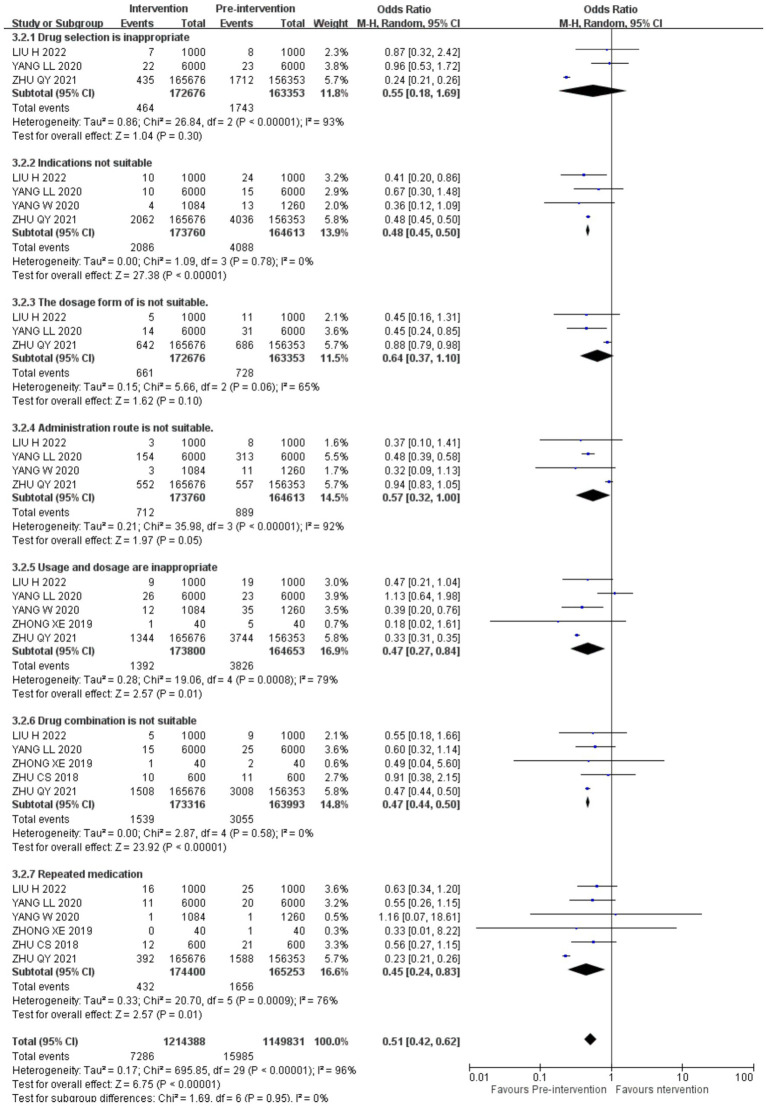
Meta-analysis results of inappropriate prescription.

The overall pooled effect size was OR = 0.51, 95%CI (0.42, 0.62), *p* < 0.00001, but with very high heterogeneity (*I*^2^ = 96%), suggesting considerable variation across and within subgroups. Major sources of heterogeneity included: the study by Zhu 2021 had a very large weight (5.7–5.8% in each subgroup), and its effect size differed markedly from those of smaller studies (e.g., for inappropriate drug selection, its OR was 0.24, while other studies had ORs near 1); in some subgroups (e.g., dosage/frequency, duplicate medication, route of administration), the direction of effect varied across studies or confidence intervals overlapped poorly; and the criteria for defining “inappropriate” as well as the intensity of interventions differed across studies. Given the very high heterogeneity (*I*^2^ = 96%), the pooled OR of 0.51 is not reliable as a precise estimate; the only firm conclusion is that the overall trend across studies favors pharmacist intervention. Results are shown in Figure 5.

#### 3.6.3 Exceptional prescriptions

The analysis of pharmacist interventions on exceptional outpatient prescriptions (off-label use without justified reason) included four studies, with a total sample size of 173,760 prescriptions in the intervention groups and 164,613 prescriptions in the pre-intervention groups. The pooled effect size was OR = 0.26, 95%CI (0.08, 0.84), *p* = 0.02, with moderate heterogeneity (*I*^2^ = 54%); we used a random-effects model. This result indicates that pharmacist intervention significantly reduces the incidence of exceptional outpatient prescriptions, with a pooled OR well below 1 and statistical significance.

**Figure 6:**
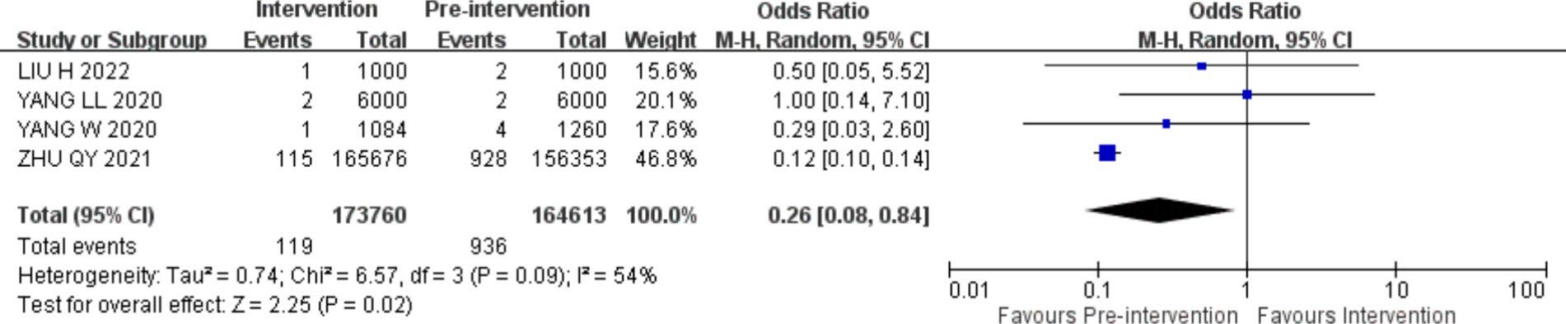
Meta-analysis results of Exceptional prescription.

The moderate heterogeneity (*I*^2^ = 54%) mainly came from differences in effect sizes across studies. The study by Zhu 2021 had an OR of 0.12 (95%CI 0.10–0.14) and accounted for 46.8% of the weight, showing a strong effect. In contrast, the studies by Liu 2022, Yang 2020, and Yang 2020 had ORs of 0.50, 1.00, and 0.29, respectively, and their confidence intervals all crossed 1, with no statistical significance and smaller sample sizes. These differences may relate to varying criteria for defining off-label use, differences in hospital management strictness, and varying intensity of pharmacist interventions. Despite some heterogeneity, the overall result appears robust, suggesting that pharmacist interventions have a clear effect in reducing off-label use without justified reason. Results are shown in Figure 6.

### 3.7 Publication bias analysis

To assess publication bias for the three core outcomes (antimicrobial prescription rationality, single-agent antimicrobial use rate, and proportion of irrational antimicrobial prescriptions), we constructed funnel plots and performed Egger’s test using Stata 14.0. All three outcomes included more than 10 studies (single-agent subgroup: 18 studies; rational prescription subgroup: 15 studies; irrational prescription subgroup: 15 studies), meeting the conditions for funnel plot and Egger’s test. Visual inspection of the funnel plots showed that, although the points did not perfectly form an inverted funnel shape, they were roughly symmetrically distributed without obvious gaps or asymmetry. Egger’s test results gave *p* values greater than 0.05 for all three outcomes: single-agent antimicrobial use (*t* = 1.51, *p* = 0.149), rational prescriptions (*t* = 1.35, *p* = 0.197), and irrational prescriptions (*t* = −1.20, *p* = 0.251). Despite the non-significant Egger‘s tests, the overall trend of positive intervention effects across all included studies (all 32 studies reported beneficial effects in the direction of improved prescribing) raises the possibility of publication bias or selective outcome reporting. Studies with null or negative findings may be less likely to be published, especially in the context of a strongly policy-driven environment in China. To further explore the potential impact of missing studies, we performed trim-and-fill analysis (using Stata 14.0) for the three core outcomes. For single-agent antimicrobial use, the trim-and-fill method imputed 3 missing studies, and the adjusted pooled OR remained 2.98 (95%CI 2.36–3.76), similar to the original estimate (3.13). For rational prescriptions, no missing studies were imputed. For irrational prescriptions, 2 missing studies were imputed, and the adjusted OR was 0.24 (95%CI 0.19–0.31), compared to the original 0.22. Detailed results are provided in Supplementary Table S5. The consistency between original and adjusted estimates suggests that while publication bias cannot be ruled out, it is unlikely to have completely reversed the conclusions.

**Figure 7:**
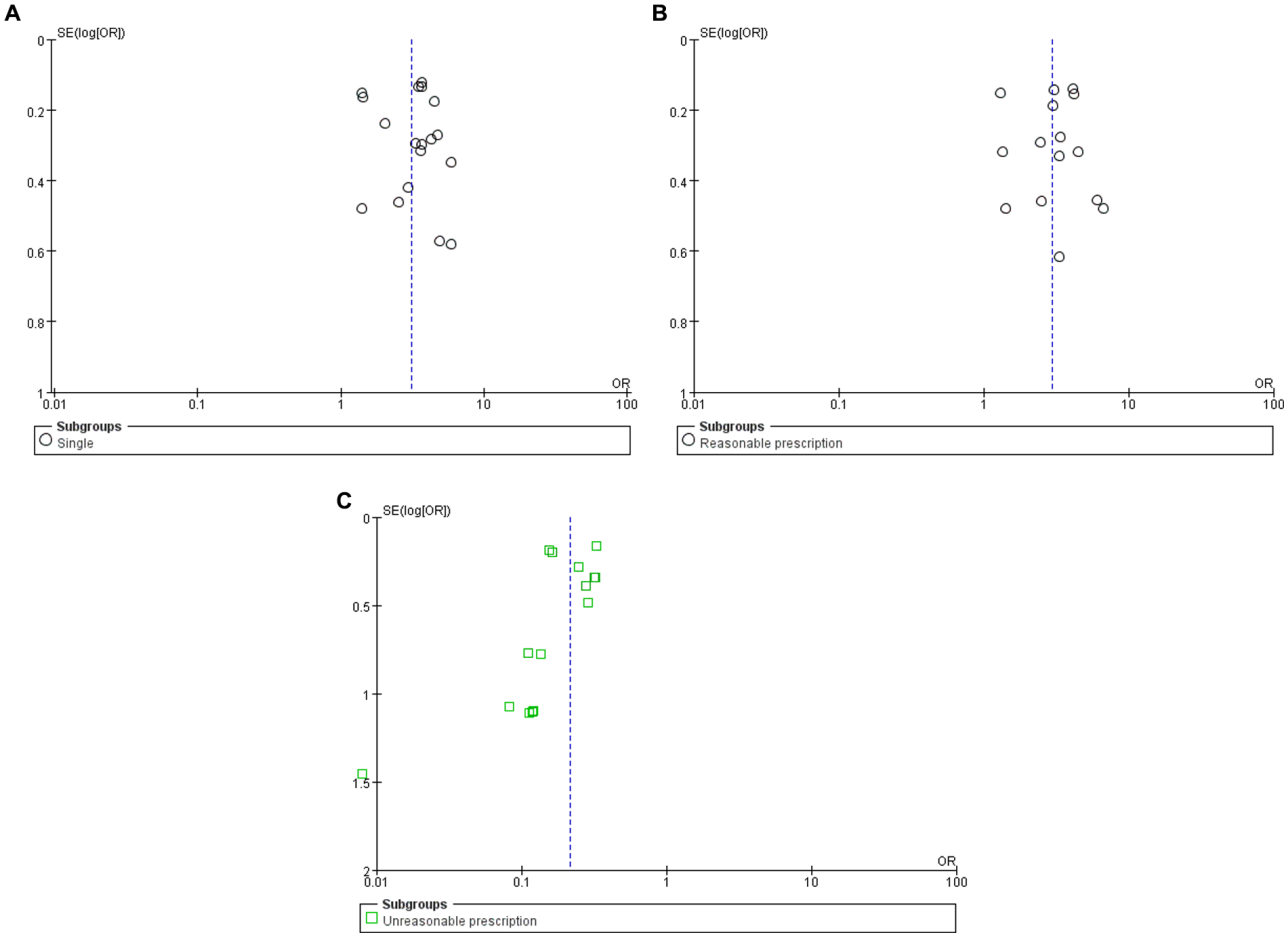
**(A)** Inverted funnel plot of comparison of combined use of antibiotics of single. **(B)** Inverted funnel plot of comparison of rationality of drug use of reasonable prescription. **(C)** Inverted funnel plot of comparison of rationality of drug use of unreasonable prescription.

These findings suggest that publication bias is absent or minimal for these three core outcome indicators, and the meta-analysis results are stable and reliable. In summary, the meta-analysis results of this study are relatively robust, with a low likelihood of being affected by publication bias. Results are shown in Figures 7A–C.

### 3.8 Subgroup analysis by study design

To explore whether study design contributed to the observed heterogeneity, we performed subgroup analyses stratified by study type (RCTs vs. NRSIs) for three core outcomes: single-agent antimicrobial use, rational antimicrobial prescriptions, and irrational antimicrobial prescriptions. The results are summarized in Supplementary Table S2.

For single-agent antimicrobial use, the pooled OR was 3.89 (95%CI 2.77–5.46, *I*^2^ = 68%) in RCTs (5 studies) and 2.96 (95%CI 2.34–3.74, *I*^2^ = 78%) in NRSIs (13 studies). For rational prescriptions, the OR was 3.52 (95%CI 2.41–5.14, *I*^2^ = 71%) in RCTs and 2.81 (95%CI 2.12–3.72, *I*^2^ = 76%) in NRSIs. For irrational prescriptions, the OR was 0.18 (95%CI 0.12–0.27, *I*^2^ = 35%) in RCTs and 0.23 (95%CI 0.18–0.30, *I*^2^ = 43%) in NRSIs. In all cases, the direction of effect was consistent between subgroups, and the confidence intervals overlapped, indicating that study design alone does not fully explain the heterogeneity. These subgroup analyses demonstrate that pooling RCTs and NRSIs did not alter the direction or significance of the effect estimates, justifying our combined analysis approach.

### 3.9 Sensitivity analyses

We performed two sensitivity analyses to further investigate heterogeneity. First, we excluded the four NRSIs rated as having high risk of bias (17, 32–34) and re-pooled the effect sizes for the three core outcomes. The results are presented in Supplementary Table S3. For irrational prescriptions, the pooled OR remained 0.24 (95%CI 0.19–0.31) and the *I*^2^ decreased from 41 to 28%. For single-agent use, the OR remained 3.07 (95%CI 2.41–3.90) with *I*^2^ decreasing from 77 to 69%. For rational prescriptions, the OR remained 2.91 (95%CI 2.22–3.81) with *I*^2^ decreasing from 75 to 68%. Exclusion of high-risk studies reduced heterogeneity modestly but did not eliminate it.

Second, we conducted leave-one-out sensitivity analysis for the single-agent antimicrobial use outcome (the highest heterogeneity, *I*^2^ = 77%). Removal of any single study did not substantially change the pooled OR (range 3.01–3.28) and the *I*^2^ remained above 70% in all iterations. This suggests that no single study was solely responsible for the high heterogeneity. Detailed results are shown in Supplementary Table S4. Collectively, these analyses indicate that the heterogeneity is likely due to true clinical diversity (e.g., differences in intervention intensity, hospital settings, and outcome definitions) rather than methodological flaws or outlier studies.

### 3.10 Sensitivity analysis using uniform outcome definitions

To assess whether definitional heterogeneity influenced the pooled estimates for antimicrobial prescription rationality, we performed a secondary meta-analysis including only studies that explicitly stated their use of the national Guiding Principles for Clinical Application of Antimicrobial Agents to define “rational” and “irrational” prescriptions. This analysis included 10 of the 15 studies reporting these outcomes. The pooled OR for rational prescriptions was 3.11 (95%CI 2.35–4.12, *I*^2^ = 73%), and for irrational prescriptions was 0.20 (95%CI 0.15–0.27, *I*^2^ = 39%). These results are similar to the main analysis (OR = 2.97 and 0.22, respectively), indicating that definitional variability did not substantially alter the direction or magnitude of the effect estimates.

### 3.11 GRADE assessment of evidence certainty

The GRADE assessment for the three core outcomes is presented in Supplementary Table S6. For single-agent antimicrobial use (18 studies, 5 RCTs and 13 NRSIs), the certainty of evidence was rated as low due to serious risk of bias (most studies were NRSIs with moderate to high risk), serious inconsistency (*I*^2^ = 77%), and suspicion of publication bias (despite non-significant Egger’s test, most studies reported positive effects). For rational antimicrobial prescriptions (15 studies), the certainty was also low for similar reasons (serious risk of bias, serious inconsistency with *I*^2^ = 75%). For irrational antimicrobial prescriptions (15 studies), the certainty was rated very low because, in addition to serious risk of bias and inconsistency, there was serious indirectness (outcome was a process measure not directly linked to patient benefit). No serious imprecision was identified for any outcome because the 95% CIs were relatively narrow and the total sample sizes exceeded the OIS. These ratings indicate that future research is likely to change the effect estimates and could potentially change the direction of the findings.

### 3.12 Sensitivity analysis excluding studies with dominant statistical weight

In the outpatient prescription analyses, the study by Zhu 2021 (35) had a very large sample size (>150,000 prescriptions) and accounted for 40–50% of the weight in several subgroups (non-standard, inappropriate, and exceptional prescriptions). To assess whether this study disproportionately influenced the pooled estimates, we performed a sensitivity analysis excluding it. The results are presented in Supplementary Table S7. For non-standard prescriptions, the pooled OR changed from 0.46 (95%CI 0.43–0.49) to 0.51 (95%CI 0.48–0.54); for inappropriate prescriptions, from 0.51 (95%CI 0.42–0.62) to 0.59 (95%CI 0.43–0.81); and for exceptional prescriptions, from 0.26 (95%CI 0.08–0.84) to 0.35 (95%CI 0.20–0.61). Heterogeneity for exceptional prescriptions decreased from *I*^2^ = 54 to 0%. These findings indicate that while the large study contributed to heterogeneity and slightly strengthened the effect estimates, the direction and general magnitude of the effects remained unchanged, supporting the robustness of the main conclusions.

## 4 Discussion

Continuous policy promotion at the national level serves as the fundamental driver for pharmacist role transformation. In 2018, the National Health Commission, together with the National Administration of Traditional Chinese Medicine, issued the Opinions on Accelerating High-Quality Development of Pharmaceutical Services, which explicitly called for accelerating the transformation of pharmaceutical service models, strengthening the pharmacy workforce, and exploring the construction of a pharmaceutical service system that meets public demand (46). Subsequently, several departments jointly released the Opinions on Strengthening Hospital Pharmacy Management to Promote Rational Drug Use, further emphasizing the need to improve pharmaceutical service standards and realize the value of pharmaceutical care (47). The core goal of these policy documents is to shift hospital pharmacy work from a drug-centered, supply-driven model toward a patient-centered, clinically oriented service model. However, a noticeable gap remains between policy direction and grassroots implementation. A study grounded in public value theory systematically analyzed the deep structure of pharmacist role fragmentation in China, pointing out a structural mismatch across institutional, social, and professional dimensions regarding pharmacists’ legal status, functional boundaries, and social recognition (48). At the same time, pharmacists’ professional self-identity shows a divided picture: some hospital pharmacists still see dispensing and inventory management as their primary tasks, while outpatient pharmacists’ depth and breadth of involvement in clinical pharmaceutical services remain at an early stage of transformation (49). All 32 studies included in this meta-analysis represent practice-based explorations of pharmaceutical service transformation in mainland Chinese medical institutions. This inclusion characteristic reflects active local efforts driven by national policies, but it also reveals, to some extent, a relative scarcity of high-quality randomized controlled trials and a predominance of pre-post self-controlled designs—a stage-specific feature of the current evidence base.

Looking at the practical outcomes of policy implementation, the meta-analysis results provide quantitative evidence supporting the positive effects of pharmacist role transformation. Our findings show that after pharmacist intervention, the proportion of single-agent antimicrobial use increased to 3.13 times that before intervention; the proportions of two-drug and three-or-more-drug combinations dropped to 0.46 and 0.24 times the pre-intervention levels, respectively; rational prescriptions rose to 2.97 times, and irrational prescriptions fell to 0.22 times. In intravenous admixture service centers, pharmacist intervention reduced the overall risk of irrational prescriptions to 0.22 times the pre-intervention level, and the incidences of inappropriate solvent selection and improper route of administration fell to 0.24 and 0.31 times, respectively. For outpatient prescription quality, non-standard prescriptions overall dropped to 0.46 times, and exceptional prescriptions to 0.26 times. The significance of these figures lies not only in statistical robustness but also in their consistency with existing international literature on pharmacist intervention effects. For instance, a systematic review and meta-analysis by Carter and colleagues (including 11 studies) showed that pharmacist-led prescription review and feedback interventions improved prescribing behavior by 22% (pooled risk ratio 0.78) (50). A Cochrane systematic review by Nkansah et al. also noted that non-dispensing pharmacist interventions in outpatient settings produced positive effects on most clinical outcomes (51). More recent international evidence supports these findings. A meta-analysis of pharmacist-led antimicrobial stewardship programs in perioperative settings demonstrated significant improvements in antibiotic selection (OR 4.29; 95% CI 2.52–7.30), administration timing (OR 4.93; 95% CI 2.05–11.84), and duration of therapy (OR 5.27; 95% CI 1.58–17.55), as well as a reduction in surgical site infections (OR 0.51; 95% CI 0.34–0.77) (52). Another systematic review focusing on pharmacist-delivered services in perioperative care found that such interventions significantly reduced hospital length of stay (SMD − 0.09; 95% CI − 0.49 to −0.15) and all-cause readmissions (OR 0.60; 95% CI 0.39–0.91) (53). Beyond the perioperative setting, pharmacist-led programs in low- and middle-income countries have been shown to reduce antimicrobial consumption, shorten therapy duration, and reduce costs (54). Community-based antimicrobial stewardship interventions also demonstrate effectiveness, albeit with considerable variation in effect sizes (55). Thus, the present study, using real-world data from China, suggests that pharmacist interventions are associated with improved prescribing process indicators, although caution is warranted given the evidence limitations. Whether these process improvements translate into clinically meaningful patient outcomes remains unknown.

Pharmacist involvement in prescription review, feedback, and order intervention serves as an institutional complement to the traditional prescribing decision-making process. From a medical management perspective, prescribing behavior is a core link in healthcare quality management and also the area where irrational drug use risks are most concentrated. In this study, pharmacist intervention was associated with a reduced incidence of inappropriate drug selection (OR 0.15), medication without indication (OR 0.35), and overly long treatment duration (OR 0.13), suggesting that embedding a pharmacist review step may help correct biases in clinical prescribing decisions. However, given the low certainty of evidence, these effects should be interpreted as exploratory. However, this “embedded oversight” of the prescribing system faces dual constraints in practice. The successful implementation of pharmacist-led interventions is influenced not only by individual professional competence but also by broader contextual factors. Implementation science frameworks highlight that barriers at multiple levels—institutional, social, and policy—can impede the adoption of new professional roles (56). For example, lack of administrative support, insufficient information technology infrastructure, and ambiguous role definitions have been identified as key implementation challenges in pharmacy practice transformation (57). Understanding these contextual determinants is essential for designing sustainable interventions and scaling pharmacist-led services from pilot projects to system-wide integration. The transformation of pharmacist roles is occurring within broader health system reforms globally. Realist evaluations of extended pharmacist roles in primary healthcare have identified multiple interacting contextual factors that enable or constrain successful role integration, including leadership commitment, infrastructure support, role clarity, and funding mechanisms (58). Similarly, community pharmacies are increasingly recognized as integral components of primary healthcare delivery, with pharmacists expanding their clinical responsibilities beyond traditional dispensing (59). These international experiences offer transferable lessons for China’s ongoing pharmacist role transformation.

On the one hand, prescribing authority rests with clinicians, and the adoption of pharmacist review opinions depends on the degree of administrative authorization and the strength of mandatory interception functions in information systems; the depth of this institutional embedding directly defines the boundaries of intervention effectiveness. On the other hand, pharmacist intervention is essentially an improvement of the internal quality control mechanism for medical care, not a substitute for external supervision. The sustainability of its long-term effects relies on the level of organizational coordination within medical institutions and the degree of support from information technology platforms. Therefore, the institutional advancement of pharmacist role transformation requires not only individual professional competence but also the establishment, at the institutional level, of standardized pharmacist-physician prescribing interaction processes and information-based prescription review support systems. These are precisely the weak links that urgently need strengthening in China’s current hospital management reform.

The substantial statistical heterogeneity observed across several pooled analyses (*I*^2^ ranging from 0 to 96%) requires careful interpretation. To explore its sources, we conducted multiple prespecified investigations: subgroup analysis by study design (RCT vs. NRSI), sensitivity analyses excluding high-risk NRSIs, leave-one-out analysis, and a sensitivity analysis restricted to studies using national outcome definitions. The subgroup analysis showed consistent effect directions between RCTs and NRSIs, indicating that study design alone did not explain the heterogeneity. Excluding the four high-risk NRSIs reduced *I*^2^ modestly (e.g., from 77 to 69% for single-agent use) but did not eliminate it. Leave-one-out analysis confirmed that no single study drove the heterogeneity. The sensitivity analysis using uniform outcome definitions produced similar effect estimates, suggesting that definitional variability was not the primary cause.

The remaining heterogeneity likely arises from true clinical and methodological diversity, including differences in intervention intensity (simple review vs. comprehensive programs), healthcare settings (outpatient vs. PIVAS, primary vs. tertiary hospitals), sample sizes (ranging from 35 to >150,000 prescriptions), and timing of outcome measurement. Consequently, while the direction of effect is consistently positive across studies, the exact pooled ORs should be interpreted with caution. The primary value of this meta-analysis is to demonstrate a consistent beneficial direction rather than to provide precise quantitative estimates that can be directly applied in clinical practice.

The outcomes synthesized in this meta-analysis are primarily process indicators (e.g., prescription rationality, combination patterns, rates of irrational prescribing). While these indicators reflect immediate improvements in prescribing behavior following pharmacist interventions, they do not directly measure patient-centric endpoints such as adverse drug reaction rates, treatment success or failure, hospital readmissions, mortality, quality of life, or healthcare costs. Improvements in prescription processes do not guarantee corresponding improvements in clinical outcomes. For example, correcting an inappropriate antimicrobial combination may reduce the risk of adverse events or bacterial resistance, but it could also delay effective treatment if not properly contextualized. A growing body of evidence suggests that process measures are valuable proxies but should be interpreted cautiously when inferring patient benefit. Future studies should incorporate clinical endpoints and health economic evaluations to establish the full value of pharmacist role transformation. The current evidence base in China lacks such outcomes, representing a major gap that warrants urgent attention in future research designs. Critically, because this meta-analysis includes only process outcomes, no conclusions can be drawn about patient-centered endpoints such as adverse drug reactions, hospital readmissions, mortality, or quality of life. Importantly, this meta-analysis provides no evidence on whether the observed improvements in prescribing processes translate into better patient-level clinical outcomes such as reduced mortality, fewer adverse drug reactions, lower hospital readmission rates, or improved quality of life. All interpretations presented herein are strictly limited to prescribing process indicators.

Several factors related to publication bias and regional bias should be considered when interpreting the positive findings of this meta-analysis. First, all included studies originated from mainland China. This geographic concentration reflects the specific policy context of pharmacist role transformation in China but also limits the generalizability of our conclusions to other healthcare systems with different pharmacist training, regulatory frameworks, and clinical cultures. Second, the vast majority of studies reported positive effects of pharmacist interventions, which may be influenced by publication bias (i.e., studies with null or negative results are less likely to be submitted or accepted for publication). Third, the strong national policy push for pharmacist role transformation during the study period (2015–2026) may have created an environment where researchers and journal editors preferentially report and accept positive findings. Although our Egger’s tests were non-significant and trim-and-fill analyses yielded similar effect estimates, these statistical methods have limited power when the number of studies is moderate and heterogeneity is high. Therefore, we cannot fully exclude the possibility that the true effect is smaller than estimated, or that the intervention is less effective in other clinical settings or countries. Readers are advised to interpret the pooled estimates as supportive but not definitive evidence of the benefits of pharmacist role transformation. Future international, multicenter studies with prospective registration and transparent reporting of all outcomes (including negative findings) are needed to confirm these results.

Using a GRADE-informed approach, the overall certainty of evidence for the primary outcomes (antimicrobial rational use, combination patterns) is low to very low. This judgment is based on: (1) the inclusion of only five RCTs; (2) the majority of NRSIs having moderate or high risk of bias on ROBINS-I; (3) substantial unexplained heterogeneity across studies; (4) indirectness of outcomes (process indicators rather than patient-centered endpoints); and (5) suspected publication bias (although statistical tests were non-significant, the predominance of positive findings is concerning). The GRADE framework, which evaluates evidence certainty across five domains (risk of bias, inconsistency, indirectness, imprecision, and publication bias), provides a standardized approach for rating confidence in effect estimates (60). Our application of GRADE followed established methodologies, and the low-to-very-low certainty ratings reflect the predominance of non-randomized designs and indirect outcomes. As formally assessed using the GRADE framework (Supplementary Table S7), the certainty of evidence for the primary outcomes was low to very low, indicating limited confidence in the effect estimates. Therefore, future research is highly likely to change the magnitude and possibly the direction of the effect estimates. Our findings are supportive of the potential benefits of pharmacist interventions on prescribing process indicators, but they do not provide conclusive evidence of effectiveness, nor do they address patient-important outcomes.

Given the predominance of non-randomized pre-post studies, the persistent high heterogeneity (*I*^2^ often > 75%), and the low-to-very-low GRADE certainty ratings, the results of this meta-analysis should be interpreted as exploratory and hypothesis-generating. They provide direction for future research but should not be taken as conclusive evidence of the effectiveness of pharmacist-led interventions in China. Confirmatory studies using rigorous randomized designs are needed before firm conclusions can be drawn.

Several pooled estimates, particularly for exceptional prescriptions (OR 0.26, 95%CI 0.08–0.84), inappropriate drug selection (OR 0.55, 95%CI 0.18–1.69), and inappropriate route of administration (OR 0.57, 95%CI 0.32–1.00), showed wide confidence intervals crossing or approaching the null, indicating imprecision due to wide confidence intervals. This imprecision reflects the small number of studies contributing to these analyses (e.g., only four studies for exceptional prescriptions), low event counts in some subgroups, and substantial between-study variability. Although the GRADE assessment did not rate imprecision as serious because the optimal information size was met, readers should recognize that the exact effect sizes for these outcomes are uncertain. Future studies with larger sample sizes and standardized outcome definitions are needed to narrow these confidence intervals.

While providing positive evidence for pharmacist transformation in China, this study also has several limitations that warrant attention in future research. First, regarding the types of included studies, constrained by the relatively small number of high-quality randomized controlled trials in China, only five of the 32 included studies were RCTs; the remaining 27 were non-randomized interventional studies, and the ROBINS-I assessment rated four of them as having high risk of bias. The overall strength of evidence needs further improvement. Second, the intervention protocols varied considerably across studies, including differences in intervention content (simple prescription review versus comprehensive interventions), intervention methods (prospective interception versus retrospective comment), implementation settings (outpatient pharmacies versus intravenous admixture centers), and study designs (pre-post self-controlled versus historical controls). These variations led to moderate to high statistical heterogeneity in several meta-analysis subgroups, requiring cautious interpretation of the pooled results. Third, the outcome measures in this study mainly focused on process indicators such as prescription quality, combination patterns, and rationality. There is a notable lack of patient-level endpoints with more direct clinical significance, including adverse drug reaction rates, treatment failure rates, hospital (re)admission rates, all-cause or disease-specific mortality, patient quality of life, and economic outcomes (e.g., cost-effectiveness, healthcare utilization). Consequently, although our findings demonstrate that pharmacist interventions improve prescribing processes, we cannot conclude with certainty that these improvements translate into better clinical outcomes for patients. Future studies should prioritize the collection of such patient-centered endpoints to provide a more complete evidence base for pharmacist role transformation in China. Fourth, the literature search was restricted to Chinese and English databases, and all included studies originated from mainland China. While this geographical focus reflects the full picture of pharmacist transformation practice in China, it also limits the direct generalizability of our conclusions to other countries or regions. Fifth, this meta-analysis pooled both RCTs and NRSIs, a practice that remains methodologically controversial. While some methodologists advise against such pooling, we adopted this approach due to the small number of available RCTs (*n* = 5) and the exploratory objective of this study. To mitigate potential bias, we performed subgroup analyses by study design and sensitivity analyses excluding high-risk NRSIs, both of which showed consistent effect directions. Nevertheless, NRSIs are inherently more susceptible to confounding and selection bias, and our unadjusted estimates may overestimate true effects. Readers should therefore interpret the pooled estimates with caution. The consistency of results across designs suggests that pooling did not produce completely misleading conclusions, but the overall evidence base would be significantly strengthened by more high-quality RCTs. Consequently, our findings should be considered hypothesis-generating rather than definitive. Sixth, although we explored heterogeneity through subgroup and sensitivity analyses, further stratification by intervention type (e.g., simple prescription review vs. comprehensive interventions) or healthcare setting (e.g., primary vs. tertiary hospitals) was not feasible due to the limited number of studies in each category. Future research should prioritize designing more high-quality randomized controlled trials, systematically collecting data on the impact of pharmacist interventions on patient clinical endpoints, and further exploring interaction effects between different intervention models and service settings, so as to more comprehensively and precisely evaluate the health economic value and overall clinical benefits of pharmacist transformation in China. Although we systematically assessed potential overlaps among studies sharing similar settings and periods, the lack of individual patient-level data prevented us from completely excluding the possibility that some prescriptions were counted in more than one study. This potential data dependency, if present, could lead to a slight overestimation of precision. However, given the diverse sources and explicit non-overlapping time windows reported by most authors, the impact on the pooled estimates is likely minimal.

## 5 Conclusion

This meta-analysis of 32 studies (5 RCTs, 27 NRSIs) provides exploratory, hypothesis-generating evidence that pharmacist-led pharmaceutical care interventions in China are associated with better prescription rationality and lower rates of antimicrobial combination therapy and other forms of irrational prescribing, but these are process outcomes only. Nevertheless, the overall quality of the included evidence is low to very low due to the small number of RCTs, the predominance of non-randomized pre-post studies with moderate to high risk of bias, persistently high statistical heterogeneity (*I*^2^ up to 92%), and the predominance of process-oriented outcomes. Therefore, these findings are exploratory and hypothesis-generating, not confirmatory. The pharmacist role transformation in China appears promising, but stronger evidence from well-designed, multicenter RCTs with patient-centered endpoints is urgently needed to establish the true clinical and economic value of these services. The pharmacist role transformation in China appears promising, but stronger evidence from well-designed, multicenter RCTs with patient-centered endpoints is urgently needed to establish the true clinical and economic value of these services. In conclusion, based on low to very low certainty evidence, pharmacist-led pharmaceutical care interventions in China are associated with improvements in prescription process indicators. However, the evidence base is weak, and high-quality RCTs are needed to confirm these findings and assess patient-important outcomes. All conclusions are restricted to prescribing process indicators; no claims about patient-level clinical outcomes are made.

## Data Availability

The datasets presented in this study can be found in online repositories. The names of the repository/repositories and accession number(s) can be found in the article/Supplementary material.
