# Supplementary material for: Effectiveness of pharmaceutical care interventions amid pharmacist role transformation in China: a meta-analysis
Source: Front Public Health. 2026 Jun 19;14:1860119. doi: 10.3389/fpubh.2026.1860119 (PMC13330479; doi:10.3389/fpubh.2026.1860119)
Supplement: Supplementary file 2 [file Table_2.docx]

**Supplementary Table S2: Subgroup analysis results by study design (RCT vs. NRSI) for three core outcomes.**

| **Outcome** | **Study design** | **No. of studies** | **Pooled OR (95% CI)** | **I²** | **P for subgroup difference** |
| --- | --- | --- | --- | --- | --- |
| Single‑agent antimicrobial use | RCT | 5 | 3.89 (2.77, 5.46) | 68% | 0.21 |
|  | NRSI | 13 | 2.96 (2.34, 3.74) | 78% |  |
| Rational antimicrobial prescriptions | RCT | 5 | 3.52 (2.41, 5.14) | 71% | 0.34 |
|  | NRSI | 10 | 2.81 (2.12, 3.72) | 76% |  |
| Irrational antimicrobial prescriptions | RCT | 5 | 0.18 (0.12, 0.27) | 35% | 0.18 |
|  | NRSI | 10 | 0.23 (0.18, 0.30) | 43% |  |

Note: RCT: randomized controlled trial; NRSI: non‑randomized interventional study. The number of studies for NRSIs is less than the total included because only studies reporting the respective outcome were included. Subgroup analyses were performed using RevMan 5.4 with a random‑effects model.

**Supplementary Table S3: Sensitivity analysis excluding high‑risk‑of‑bias studies.**

| **Outcome** | **Analysis** | **No. of studies** | **Pooled OR (95% CI)** | **I²** |
| --- | --- | --- | --- | --- |
| Single‑agent antimicrobial use | Original (all studies) | 18 | 3.13 (2.49, 3.92) | 77% |
|  | After excluding 4 high‑risk NRSIs* | 14 | 3.07 (2.41, 3.90) | 69% |
| Rational antimicrobial prescriptions | Original (all studies) | 15 | 2.97 (2.30, 3.85) | 75% |
|  | After excluding 4 high‑risk NRSIs* | 11 | 2.91 (2.22, 3.81) | 68% |
| Irrational antimicrobial prescriptions | Original (all studies) | 15 | 0.22 (0.17, 0.28) | 41% |
|  | After excluding 4 high‑risk NRSIs* | 11 | 0.24 (0.19, 0.31) | 28% |

Note: *High‑risk NRSIs (ROBINS‑I overall rating = high): Zhang JX 2015 [17], Che HY 2021 [32], Huang LP 2024 [33], Zhang JL 2021 [34]. After exclusion, the remaining studies were all either RCTs (5) or moderate‑risk NRSIs (9 for single‑agent; 6 for rational/irrational).

**Supplementary Table S4: Leave‑one‑out sensitivity analysis for single‑agent antimicrobial use.**

| **Study omitted** | **Pooled OR (95% CI) after omission** | **I² after omission (%)** |
| --- | --- | --- |
| Chen GR 2018 [9] | 3.09 (2.44, 3.92) | 76 |
| Zong CL 2018 [10] | 3.11 (2.45, 3.95) | 76 |
| Zhou XF 2019 [11] | 3.14 (2.47, 3.98) | 77 |
| Gao LJ 2019 [12] | 3.12 (2.46, 3.96) | 76 |
| Guo J 2018 [14] (RCT) | 3.01 (2.38, 3.81) | 74 |
| Wu GF 2018 [15] | 3.15 (2.49, 3.99) | 77 |
| Liao SX 2019 [16] | 3.10 (2.44, 3.94) | 76 |
| Zhang JX 2015 [17] (high risk) | 3.08 (2.43, 3.90) | 75 |
| Wang GQ 2022 [18] (RCT) | 3.28 (2.58, 4.17) | 78 |
| Wang YD 2019 [20] | 3.13 (2.46, 3.98) | 77 |
| Geng AL 2019 [21] | 3.11 (2.45, 3.95) | 76 |
| Jia YY 2019 [22] | 3.14 (2.47, 3.99) | 77 |
| Chen DE 2019 [23] | 3.09 (2.44, 3.92) | 76 |
| Yang YL 2018 [24] | 3.10 (2.44, 3.94) | 76 |
| Zou JJ 2020 [25] | 3.16 (2.50, 4.00) | 78 |
| Zhuo FY 2023 [26] | 3.12 (2.46, 3.96) | 76 |
| Tang JY 2019 [27] (RCT) | 3.18 (2.52, 4.02) | 78 |
| Yao Y 2019 [29] | 3.13 (2.46, 3.98) | 77 |

Note: No single study omission changed the pooled OR substantially (range 3.01–3.28) or reduced the I² below 70%, indicating that the high heterogeneity is not driven by any individual study but reflects genuine clinical diversity. RCTs = randomized controlled trials; high‑risk = studies with overall high risk of bias according to ROBINS‑I.

**Supplementary Table S5：Trim‑and‑fill analysis results for three core outcomes**

| **Outcome** | **Original OR (95% CI)** | **Imputed missing studies (n)** | **Adjusted OR (95% CI)** |
| --- | --- | --- | --- |
| Single‑agent antimicrobial use | 3.13 (2.49–3.92) | 3 | 2.98 (2.36–3.76) |
| Rational antimicrobial prescriptions | 2.97 (2.30–3.85) | 0 | 2.97 (2.30–3.85) |
| Irrational antimicrobial prescriptions | 0.22 (0.17–0.28) | 2 | 0.24 (0.19–0.31) |

Note: Trim‑and‑fill analysis was performed using Stata 14.0 with the random‑effects model. The imputed studies are hypothetical missing studies assumed to be symmetrically distributed on the opposite side of the funnel plot.

**Supplementary Table S6. GRADE assessment of evidence certainty for core outcomes**

| **Outcome** | **No. of studies (design)** | **Risk of bias** | **Inconsistency** | **Indirectness** | **Imprecision** | **Publication bias** | **Overall certainty** | **Importance** |
| --- | --- | --- | --- | --- | --- | --- | --- | --- |
| Single‑agent antimicrobial use (OR >1 indicates improvement) | 18 (5 RCTs, 13 NRSIs) | Serious¹ | Serious² (I²=77%) | Not serious³ | Not serious⁴ | Suspected⁵ | Low | Critical |
| Rational antimicrobial prescriptions (OR >1 indicates improvement) | 15 (5 RCTs, 10 NRSIs) | Serious¹ | Serious² (I²=75%) | Not serious³ | Not serious⁴ | Suspected⁵ | Low | Critical |
| Irrational antimicrobial prescriptions (OR <1 indicates improvement) | 15 (5 RCTs, 10 NRSIs) | Serious¹ | Serious² (I²=41%) | Serious⁶ | Not serious⁴ | Suspected⁵ | Very low | Critical |

**Explanations:**
¹ **Serious risk of bias**: Only 5 of 32 studies were RCTs; most NRSIs had moderate or high risk of bias on ROBINS‑I (23 moderate, 4 high). Allocation concealment and blinding were often unclear in RCTs.
² **Serious inconsistency**: Substantial statistical heterogeneity (I² > 75% for two outcomes, 41% for the third) and variable effect sizes across studies, despite consistent direction.
³ **Not serious indirectness**: Prescription rationality is a direct measure of the intervention’s immediate target, even if not a patient‑centered outcome.
⁴ **Not serious imprecision**: The 95% confidence intervals were relatively narrow, and the total sample sizes exceeded the optimal information size (OIS) for all outcomes.
⁵ **Suspected publication bias**: All studies reported positive effects; funnel plots showed slight asymmetry although Egger’s tests were non‑significant.
⁶ **Serious indirectness**: Irrational prescription rate is a process indicator that does not directly measure patient health outcomes (e.g., adverse events, treatment success).

**GRADE interpretation:**

- **Low certainty**: Further research is very likely to have an important impact on our confidence in the estimate of effect and is likely to change the estimate.
- **Very low certainty**: Any estimate of effect is very uncertain.

**Supplementary Table S7. Domain‑level ROBINS‑I assessments for included non‑randomized studies (NRSIs)**

| **Study ID** | **Confounding** | **Selection of participants** | **Classification of interventions** | **Deviations from intended interventions** | **Missing data** | **Measurement of outcomes** | **Selective reporting** | **Overall** | **Justification summary** |
| --- | --- | --- | --- | --- | --- | --- | --- | --- | --- |
| Chen GR 2018 [9] | Moderate | Low | Low | Moderate | Low | Moderate | Low | Moderate | No adjustment for baseline differences; outcome assessors not blinded |
| Zong CL 2018 [10] | Moderate | Low | Low | Moderate | Low | Moderate | Low | Moderate | Same as above |
| Zhou XF 2019 [11] | Moderate | Low | Low | Low | Low | Moderate | Low | Moderate | Pre‑post design, no adjustment |
| Gao LJ 2019 [12] | Moderate | Low | Low | Moderate | Low | Moderate | Low | Moderate | Baseline differences not accounted for |
| Hu DM 2020 [13] | Moderate | Low | Low | Moderate | Low | Moderate | Low | Moderate | Same pattern |
| Wu GF 2018 [15] | Moderate | Low | Low | Moderate | Low | Moderate | Low | Moderate | No confounder control |
| Liao SX 2019 [16] | Moderate | Low | Low | Moderate | Low | Moderate | Low | Moderate | – |
| Zhang JX 2015 [17] | High | Moderate | Low | High | Moderate | Moderate | Low | High | No baseline comparability; intervention fidelity unclear |
| Deng GP 2019 [19] | Moderate | Low | Low | Moderate | Low | Moderate | Low | Moderate | – |
| Wang YD 2019 [20] | Moderate | Low | Low | Moderate | Low | Moderate | Low | Moderate | – |
| Geng AL 2019 [21] | Moderate | Low | Low | Moderate | Low | Moderate | Low | Moderate | – |
| Jia YY 2019 [22] | Moderate | Low | Low | Moderate | Low | Moderate | Low | Moderate | – |
| Chen DE 2019 [23] | Moderate | Low | Low | Moderate | Low | Moderate | Low | Moderate | – |
| Yang YL 2018 [24] | Moderate | Low | Low | Moderate | Low | Moderate | Low | Moderate | – |
| Zou JJ 2020 [25] | Moderate | Low | Low | Moderate | Low | Moderate | Low | Moderate | – |
| Zhuo FY 2023 [26] | Moderate | Low | Low | Low | Low | Moderate | Low | Moderate | – |
| Geng LL 2019 [28] | Moderate | Low | Low | Moderate | Moderate | Moderate | Low | Moderate | Some missing data but not critical |
| Yao Y 2019 [29] | Moderate | Low | Low | Moderate | Low | Moderate | Low | Moderate | – |
| Cai W 2025 [30] | Moderate | Low | Low | Moderate | Low | Moderate | Low | Moderate | – |
| Gu DM 2019 [31] | Moderate | Low | Low | Moderate | Moderate | Moderate | Low | Moderate | Some missing data |
| Che HY 2021 [32] | High | Moderate | Low | High | High | Moderate | Low | High | No adjustment; large missing data; blinding absent |
| Huang LP 2024 [33] | High | Moderate | Low | High | Moderate | Moderate | Low | High | No confounder control; intervention integrity unclear |
| Zhang JL 2021 [34] | High | Moderate | Low | High | Moderate | Moderate | Low | High | Same pattern as above |
| Zhu QY 2021 [35] | Moderate | Low | Low | Moderate | Low | Moderate | Low | Moderate | – |
| Zhu CS 2018 [37] | Moderate | Low | Low | Moderate | Low | Moderate | Low | Moderate | – |
| Liu H 2022 [38] | Moderate | Low | Low | Moderate | Low | Moderate | Low | Moderate | – |
| Yang W 2020 [39] | Moderate | Low | Low | Moderate | Low | Moderate | Low | Moderate | – |

Note: Risk levels are defined per ROBINS‑I. “Moderate” in this table corresponds to the “moderate risk” category in the main text. Justifications are summarized; full signaling question responses are available from the corresponding author upon request.
